# Neuronal LRP4 directs the development, maturation and cytoskeletal organization of *Drosophila* peripheral synapses

**DOI:** 10.1242/dev.202517

**Published:** 2024-06-03

**Authors:** Alison T. DePew, Joseph J. Bruckner, Kate M. O'Connor-Giles, Timothy J. Mosca

**Affiliations:** ^1^ Department of Neuroscience, Vickie and Jack Farber Institute of Neuroscience, Thomas Jefferson University, Philadelphia, PA 19107, USA; ^2^ Cell and Molecular Biology Training Program, University of Wisconsin-Madison, Madison, WI 53706, USA; ^3^ Department of Neuroscience, Brown University, Providence, RI 02912, USA; ^4^ Carney Institute for Brain Science, Brown University, Providence, RI 02912, USA

**Keywords:** LRP4, SRPK79D, Synapse, Neuromuscular junction, *Drosophila*, Synapse development

## Abstract

Synaptic development requires multiple signaling pathways to ensure successful connections. Transmembrane receptors are optimally positioned to connect the synapse and the rest of the neuron, often acting as synaptic organizers to synchronize downstream events. One such organizer, the LDL receptor-related protein LRP4, is a cell surface receptor that has been most well-studied postsynaptically at mammalian neuromuscular junctions. Recent work, however, identified emerging roles, but how LRP4 acts as a presynaptic organizer and the downstream mechanisms of LRP4 are not well understood. Here, we show that LRP4 functions presynaptically at *Drosophila* neuromuscular synapses, acting in motoneurons to instruct pre- and postsynaptic development. Loss of presynaptic LRP4 results in multiple defects, impairing active zone organization, synapse growth, physiological function, microtubule organization, synaptic ultrastructure and synapse maturation. We further demonstrate that LRP4 promotes most aspects of presynaptic development via a downstream SR-protein kinase, SRPK79D. These data demonstrate a function for presynaptic LRP4 as a peripheral synaptic organizer, highlight a downstream mechanism conserved with its CNS function in *Drosophila*, and underscore previously unappreciated but important developmental roles for LRP4 in cytoskeletal organization, synapse maturation and active zone organization.

## Teaser

**Summary:** LRP4 is required in neurons to instruct diverse aspects of synapse development at the *Drosophila* neuromuscular junction, highlighting previously unknown presynaptic roles for this receptor at peripheral synapses.

## INTRODUCTION

The successful development of a synapse is intricate, requiring the coordination of diverse molecular players. Upon contact, pre- and postsynaptic partner neurons undergo additional growth, transcriptional changes, structural and cytoskeletal remodeling, and specialized synaptic protein recruitment (Harris and Littleton, 2015). The series of complex processes at connections is crucial for the reliable function of the synapse. Consequently, synapse development errors can disrupt neuronal activity and underlie neurodevelopmental, neuropsychiatric and neurodegenerative disorders, including autism, epilepsy and schizophrenia (Barnat et al., 2020; Bennett, 2011; Bonansco and Fuenzalida, 2016; Gilbert and Man, 2017; Grant, 2012; Melom and Littleton, 2011). Given the importance of precision in synapse formation, multiple developmental steps must be carefully coordinated, often by ‘synaptic organizer’ proteins (Li and Sheng, 2003; Scheiffele, 2003; Siddiqui and Craig, 2011). Understanding the identity of, and the pathways by which, synaptic organizers coordinate synaptogenesis is a crucial step towards understanding the development and dysregulation of the nervous system.

The low-density lipoprotein-related receptor LRP4 acts as a synaptic organizer in invertebrates and vertebrates (DePew and Mosca, 2021). As a cell-surface receptor, LRP4 is optimally positioned to signal across the synapse during development and to instruct downstream events. In its most well-studied role in mammalian neuromuscular junction (NMJ) formation, LRP4 is the postsynaptic receptor for Agrin and initiates a cascade of events, beginning with phosphorylation of the kinase MuSK (Kim et al., 2008; Weatherbee et al., 2006; Zhang et al., 2008). MuSK phosphorylation leads to subsequent phosphorylation of Dok7 (Beeson et al., 2006; Okada et al., 2006), followed by recruitment and clustering of postsynaptic proteins, such as acetylcholine receptors, and activation of synapse-specific transcription (Burden et al., 2013). Following synapse formation, LRP4 acts to maintain connections; this post-developmental role underlies its implication in motor unit disorders such as amyotrophic lateral sclerosis and myasthenia gravis (Barik et al., 2014; Pevzner et al., 2012; Rivner et al., 2017; Tzartos et al., 2014; Zhang et al., 2012; Zhao et al., 2018).

Despite intense study, presynaptic LRP4 function remains far less examined. In the *Drosophila* CNS, presynaptic LRP4 regulates excitatory active zone number and function via the SR-protein kinase SRPK79D. As *Drosophila* lack clear Agrin and MuSK homologues (Mosca et al., 2017), LRP4 must act independently of such factors. But how LRP4 in the fly acts trans-synaptically, or in other stages of synaptic organization, development and maturation are unknown. Whether mammalian LRP4 functions presynaptically (either in the CNS or at the NMJ) also remains unknown. Recent evidence posits LRP4 as a regulator of CNS synapse number and dendrite morphology, although where it functions is currently inconclusive (Gomez et al., 2014; Handara et al., 2019; Karakatsani et al., 2017). Furthermore, recent evidence suggests LRP4 in the mammalian CNS is almost exclusively glial (Karlsson et al., 2021), suggesting multiple mechanisms may underlie LRP4 function *in toto*. There is some genetic evidence of a motoneuron role for LRP4 at the mammalian NMJ (Wu et al., 2012) but most data indicate postsynaptic function. Taken together, studies suggest LRP4 is an important player in general synapse development (DePew and Mosca, 2021) but its precise functional mechanisms remain unknown. *Drosophila* is a uniquely positioned and advantageous system for assessing presynaptic LRP4 function and such mechanisms, as critical open questions remain regarding how LRP4 functions as a synaptic organizer: Does LRP4 function presynaptically at synapses? What stages of synaptic development require LRP4? What cellular processes does LRP4 instruct during neurodevelopment? What are the downstream effectors of LRP4 in promoting synapse organization?

**Fig. 1.**
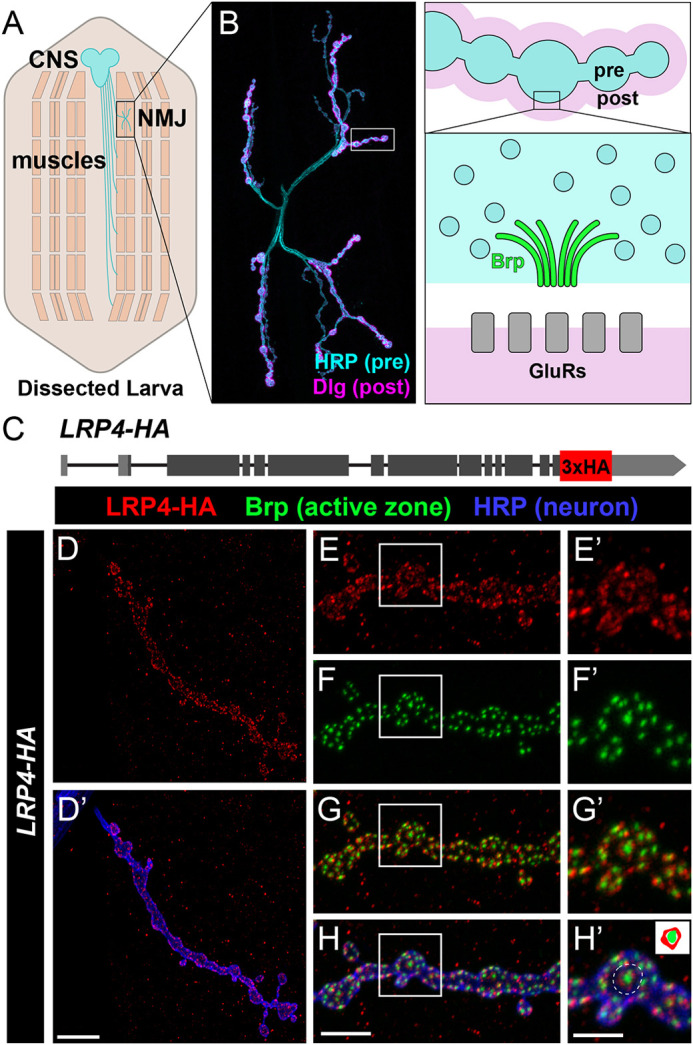
**LRP4 localizes near active zones and is required for active zone organization.** (A) Schematic of a *Drosophila* third instar larva. Motor axons from the central nervous system (blue) synapse with muscle fibers (tan) that are organized in repeating segments. (B) Representative confocal image (left) of a wild-type NMJ stained for HRP (cyan) to visualize neuronal membranes, and Dlg (magenta) to mark postsynapses. A diagram (right) depicting presynaptic boutons (blue) surrounded by postsynaptic membrane (pink). The enlargement depicts the active zone and highlights the active zone protein Bruchpilot (green) that apposes postsynaptic glutamate receptors (gray). (C) Diagram of the *lrp4* genomic region with a 3xHA tag inserted at the C terminus. (D) Representative confocal image of a NMJ expressing endogenous LRP4-HA, stained with antibodies to HA (red) and HRP (blue). LRP4-HA expression is visible within the motoneuron. Scale bar: 10 µm. (E-H) Representative confocal image of an NMJ expressing endogenous LRP4-HA, stained with antibodies to HA (red), HRP (blue) and Brp (green). The outlined areas are shown at higher magnification on the right. LRP4-HA staining is punctate and surrounds Brp-positive active zones. Inset in H′ depicts LRP4 staining surrounding an active zone, as observed in the region surrounded by a dotted line. Scale bars: 5 µm in E-H; 2.5 µm in E′-H′.

The glutamatergic *Drosophila* larval neuromuscular junction (NMJ) is a powerful genetic system for dissecting the pre- and postsynaptic cellular mechanisms underlying synapse development (Fig. 1A) with translational potential. Precise genetic tools and single-synapse imaging capability allow careful developmental investigation through cell-type specific analysis (Chou et al., 2020a; Collins and DiAntonio, 2007; Harris and Littleton, 2015; Keshishian et al., 2003). NMJ development can be categorized by multiple overlapping stages, including synapse growth, cytoskeletal reorganization, active zone assembly and maturation (Chou et al., 2020a). The precise pre- and postsynaptic roles of individual genes in each of these processes can be readily dissected, offering the opportunity to study how synaptic organizers such as LRP4 influence diverse phases of synapse organization and their underlying cellular mechanisms. Despite roles in CNS axon targeting and olfaction (Douthit et al., 2021; Mosca et al., 2017), how LRP4 regulates fly development is not well understood. To begin to address this knowledge gap, we used the *Drosophila* NMJ to study synaptic LRP4. Here, we demonstrate that LRP4 is expressed in motoneurons and localizes near active zones at the developing NMJ; we find that LRP4 acts cell-autonomously in presynaptic motoneurons to regulate nearly all aspects of synaptic development, including active zone organization, synaptic function, NMJ growth and cytoskeletal structure. We also observe that LRP4 is required for postsynaptic maturation, as loss of *lrp4* (FBgn0030706) impairs recruitment of postsynaptic scaffolding proteins and formation of the postsynaptic spectrin cytoskeleton. LRP4 functions presynaptically to regulate synaptic maturation, suggesting cell-autonomous and cell non-autonomous roles. Finally, we demonstrate that LRP4 acts genetically via the downstream SR-protein kinase SRPK79D in motoneurons to regulate development. Our work highlights the importance of LRP4 as a synaptic organizer that directs multiple cellular processes during development, and reveals previously unreported roles for LRP4 in regulating microtubule organization and synapse maturation independently of Agrin/MuSK. These findings suggest a novel presynaptic LRP4-dependent action at *Drosophila* synapses that can further inform how we understand synapse formation.

## RESULTS

### LRP4 is expressed presynaptically near active zones at the NMJ

In *Drosophila*, *lrp4* transcript expression is detected in motoneurons (Li et al., 2022) but this neither confirms protein expression nor provides the resolution needed to determine whether LRP4 functions presynaptically at the NMJ or postsynaptically to interneuron populations. Thus, we first determined whether LRP4 protein is expressed in motoneurons and identified where LRP4 localizes. We confirmed LRP4 expression at the presynaptic NMJ by driving GFP using an *lrp4-GAL4* driver (Mosca et al., 2017; Pfeiffer et al., 2008) and observed GFP in motoneurons (Fig. S1A). To determine where LRP4 protein localizes, we used CRISPR-Cas9 genome editing (Gratz et al., 2013; 2014) to generate LRP4-HA, a fly line in which endogenous LRP4 is tagged with a 3x-HA epitope at the C-terminus (Fig. 1C), as C-terminal tagging does not block function (Mosca et al., 2017). LRP4-HA flies were viable and showed no overt phenotypes in neuronal morphology or changes to bouton number, indicating the tag did not abrogate normal function (Fig. S2). We observed LRP4-HA staining in motoneurons (Fig. 1D) – supporting our GFP expression data – in a punctate pattern within presynaptic NMJ boutons (Fig. 1E-H). Co-staining with the active zone marker Bruchpilot (Brp) showed LRP4-HA localization near active zones, often surrounding Brp puncta (Fig. 1E-H). To independently confirm our findings, we also expressed a tagged *lrp4* transgene, *UAS-LRP4-HA*, in neurons via UAS/GAL4, and observed similar HA staining within boutons (Fig. S1B) and synaptic localization. Taken together, these data demonstrate that LRP4 protein is expressed in motoneurons and localizes presynaptically at the NMJ, suggesting that it may act developmentally and functionally near the active zone.

### Perturbation of *lrp4* affects active zone organization and function

**Fig. 2.**
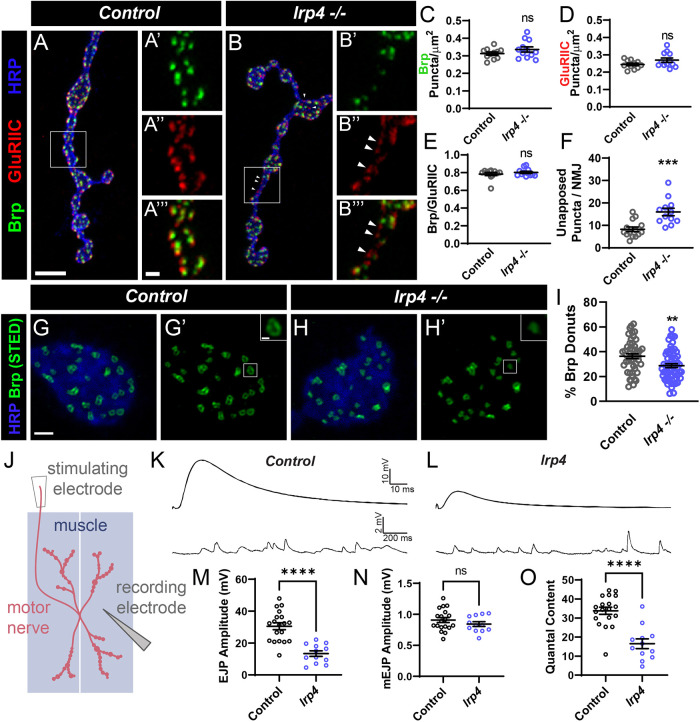
**Loss of *lrp4* leads to defects in active zone apposition and function.** (A-B‴) Representative confocal images of control (A-A‴) and *lrp4* mutant (B-B‴) NMJs stained with antibodies to Brp (green), GluRIIC (red) and HRP (blue). Arrowheads indicate unapposed puncta. Scale bars: 5 µm in A,B; 2 µm in A′-A‴, B′-B‴. (C) Quantification of Brp density. (D) Quantification of GluRIIC density. (E) Quantification of the ratio of Brp to GluRIIC puncta. (F) Quantification of unapposed puncta per NMJ. (G-H′) Representative images of control (G,G′) and *lrp4* mutant (H,H′) NMJs stained with antibodies to HRP (blue) and Brp (green) visualized with STED microscopy. Insets show an example of a donut-shaped punctum. Scale bars: 1 µm; 200 nm in insets. (I) Quantification of the percentage of donut-shaped Brp puncta (with a center hole). (J) Diagram of experimental electrophysiology setup. An electrode records from a muscle fiber in the presence (to record EJPs) or absence (to record mEJPs) of motor axon stimulation. (K,L) Representative EJP and mEJP traces recorded from wild-type (K) and *lrp4^Del^/y* (L) muscle. (M) Quantification of EJP amplitude. (N) Quantification of mEJP amplitude. (O) Quantification of quantal content. For all experiments, data are mean±s.e.m. **P<0.01, ***P<0.001, ****P<0.0001; ns, not significant. Significance was determined using an unpaired two-tailed Student's *t*-test. (C-F) *n*≥12 NMJs, *n*=6 larvae. (I) *n*≥47 boutons, *n*=8 larvae. (M-O) *n*≥12 NMJs, *n*=5 larvae.

Active zones consist of a host of proteins surrounding a central cluster of calcium channels, promoting Ca^2+^-regulated neurotransmitter release (Van Vactor and Sigrist, 2017). As we observe LRP4 presynaptically near active zones, we reasoned that LRP4 may be involved in active zone organization. To test our hypothesis, we disrupted LRP4 function using a previously generated null mutant (*lrp4^dalek^*) lacking the *lrp4*-coding region (Mosca et al., 2017). At the NMJ, the active zone scaffolding protein Brp (the ortholog of vertebrate ELKS/CAST) regulates neurotransmission and active zone structure (Wagh et al., 2006), and is closely apposed to postsynaptic glutamate receptor tetramers containing the obligate GluRIIC/DGluRIII subunit (Collins and DiAntonio, 2007; Marrus et al., 2004) (Fig. 1B). To first determine whether LRP4 influences active zone and glutamate receptor cluster density, we stained for Bruchpilot and GluRIIC, and quantified pre- and postsynaptic puncta (Fig. 2A,B). Loss of *lrp4* did not alter the density of Brp (Fig. 2C) or GluRIIC (Fig. 2D) puncta nor the ratio of Brp puncta to GluRIIC puncta (Fig. 2E). However, we did observe a significant increase in the number of unapposed active zones and receptor clusters per NMJ, defined as Brp puncta lacking apposite GluRIIC puncta, or vice versa (Fig. 2F). Unapposed GluRIIC puncta accounted for 80% of the apposition errors in the *lrp4* mutant (Fig. S3A,B). This increase in unapposed puncta suggests that, although *lrp4* does not influence active zone density, it promotes normal active zone apposition.

Although active zone apposition changes were not accompanied by changes in density, we could not rule out the possibility that LRP4 influenced the organization of individual active zones. Via confocal imaging, active zones appear as individual puncta (Wagh et al., 2006) but when imaged using super-resolution stimulated emission depletion (STED) microscopy, Brp puncta appear as donut-like structures in type Ib boutons when oriented planar to the imaging axis (Fouquet et al., 2009; Jetti et al., 2023; Kittel et al., 2006). Defects in synaptic organization are often accompanied by changes to this donut structure (Barber et al., 2018; Bruckner et al., 2017; Jetti et al., 2023; Liu et al., 2011). Using STED microscopy, we observed multiple Bruchpilot-positive active zone puncta with clear donut-shaped morphology in wild-type larvae (Fig. 2G). We also observe a proportion of puncta in wild-type boutons that lack a center hole; these likely represent donut-shaped puncta observed laterally (Fig. 2G). In *lrp4* mutants, we observed significantly fewer puncta that could be resolved as a donut (Fig. 2H-I), further suggesting that LRP4 is required for the organization of individual active zones.

To determine whether the active zone defects we observed corresponded to functional deficits, we measured neurotransmission in NMJs lacking *lrp4.* We recorded both spontaneous and evoked potentials from muscles of wild-type and *lrp4* mutant larvae (Fig. 2J-L). Loss of *lrp4* caused a 56% decrease in the amplitude of excitatory junctional potentials (EJPs), indicating impaired neurotransmission (Fig. 2K-M). Loss of *lrp4* did not affect the amplitude (Fig. 2K,L,N) of spontaneous miniature EJPs (mEJPs), suggesting the defect was not solely postsynaptic. We calculated quantal content and determined that neurotransmitter release is significantly reduced in the absence of *lrp4* (Fig. 2O). Together, our data indicate that LRP4 promotes the function, organization and apposition of individual active zones, suggesting LRP4 is required for proper synaptic development.

### Neuronal LRP4 is critical for NMJ growth and microtubule organization

**Fig. 3.**
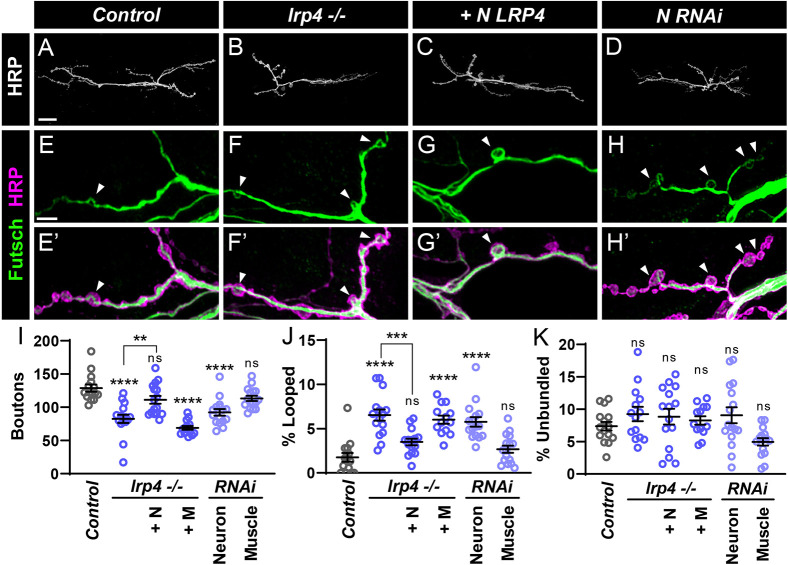
**Motoneuron LRP4 is required for synapse growth and microtubule organization.** (A-D) Representative confocal images of NMJs from control (A), *lrp4* mutant (B), *lrp4* mutant expressing LRP4 in motoneurons (C) and *lrp4* motoneuron RNAi (D) larvae stained with antibodies to HRP. Scale bar: 25 µm. (E-H′) Representative confocal images of NMJs from control (E,E′), *lrp4* mutant (F,F′), *lrp4* mutant expressing LRP4 in motoneurons (G,G′) and *lrp4* motoneuron RNAi (H,H′) larvae stained with antibodies to Futsch (green) and HRP (magenta). Arrowheads indicate Futsch loops. Scale bar: 5 µm. (I) Quantification of bouton number from experiments in A-D. +N, expression in motoneurons; +M, expression in muscle. (J) Quantification of the percentage of boutons containing looped Futsch, from experiments in E-H′. +N, expression in motoneurons; +M, expression in muscle. (K) Quantification of the percentage of boutons containing unbundled Futsch, +N, expression in motoneurons; +M, expression in muscle. For all experiments, data are mean±s.e.m. **P<0.01, ***P<0.001, ****P<0.0001, ns, not significant. Significance was calculated using one-way ANOVA, followed by Tukey's test for multiple comparisons. *n*≥13 NMJs, *n*=8 larvae.

We next sought to determine whether loss of *lrp4* affects synapse growth beyond active zones. We assessed overall NMJ arborization (Fig. 3A,B) and observed a 35% decrease in synaptic bouton number after *lrp4* loss (Fig. 3I)*.* To determine where *lrp4* acts to regulate bouton number, we performed tissue-specific rescue experiments in *lrp4* mutants and found that LRP4 expressed in motoneurons (Fig. 3C), but not muscles (Fig. S4B) or glia (Fig. S5A-C), is sufficient to rescue the bouton phenotype (Fig. 3I). Furthermore, *lrp4* RNAi in motoneurons (Fig. 3D), but not muscle (Fig. S4C), recapitulates the reduction in bouton number (Fig. 3I). Interestingly, neuronal overexpression of LRP4 increased bouton number (Fig. S6A-C), suggesting that motoneuron LRP4 can act instructively to control neuronal arborization and synapse formation. These data indicate that presynaptic LRP4 is required beyond active zones for normal NMJ growth.

At the *Drosophila* NMJ, microtubule perturbations often accompany synaptic growth defects (Menon et al., 2013; Pennetta et al., 2002; Roos et al., 2000; Ruiz-Canada et al., 2004). As such, the microtubule-associated protein Futsch/MAP1B acts in motoneurons to promote synaptic growth and active zone stabilization (Hummel et al., 2000; Lepicard et al., 2014; Roos et al., 2000). As a potential mechanism underlying the disrupted growth in *lrp4* mutants, we examined cytoskeletal organization using Futsch staining to visualize microtubules (Fig. 3E-H). In wild-type larvae, Futsch staining reveals branches of microtubules throughout the terminal, with looped or unbundled structures present within boutons. Loops are thought to indicate microtubule stability (Nechipurenko and Broihier, 2012; Roos et al., 2000; Shi et al., 2019), which may correlate with restricted growth (Chou et al., 2020b). Conversely, unbundled, diffuse Futsch is typically associated with disorganized microtubules (Roos et al., 2000). *lrp4* mutants show an over threefold increase in the percentage of boutons containing loops (Fig. 3F,J), despite a 35% reduction in the total number of boutons (Fig. 3I). This increase in looped Futsch is suppressed by expression of LRP4 in motoneurons (Fig. 3G,J), but not in muscles (Fig. S4E). Conversely, knockdown of *lrp4* in motoneurons increases loops (Fig. 3H), whereas muscle knockdown has no effect (Fig. S4F, Fig. 3J). We also quantified boutons containing unbundled Futsch in all genotypes and observed no significant differences (Fig. 3K). The observed changes in Futsch indicate that presynaptic LRP4 regulates microtubule organization, and results in abnormally stabilized microtubules, which may contribute to *lrp4* mutant-associated growth defects.

### LRP4 in motoneurons is required for synapse maturation

After the initial stages of growth, bouton addition and active zone assembly, the synapse matures into a stable connection. During synapse maturation, boutons recruit postsynaptic glutamate receptors (Schmid et al., 2008), scaffolding proteins (Ataman et al., 2006; Packard et al., 2002) and cytoskeletal elements (Restrepo et al., 2022) to ensure lasting strength. Impaired synapse maturation is marked by an increase in the number of immature boutons - termed ‘ghost boutons’ - that contain presynaptic membrane but lack apposite postsynaptic markers, such as Dlg (Ataman et al., 2006), and show reduced spectrin surrounding the bouton (Mosca and Schwarz, 2010; Restrepo et al., 2022).

**Fig. 4.**
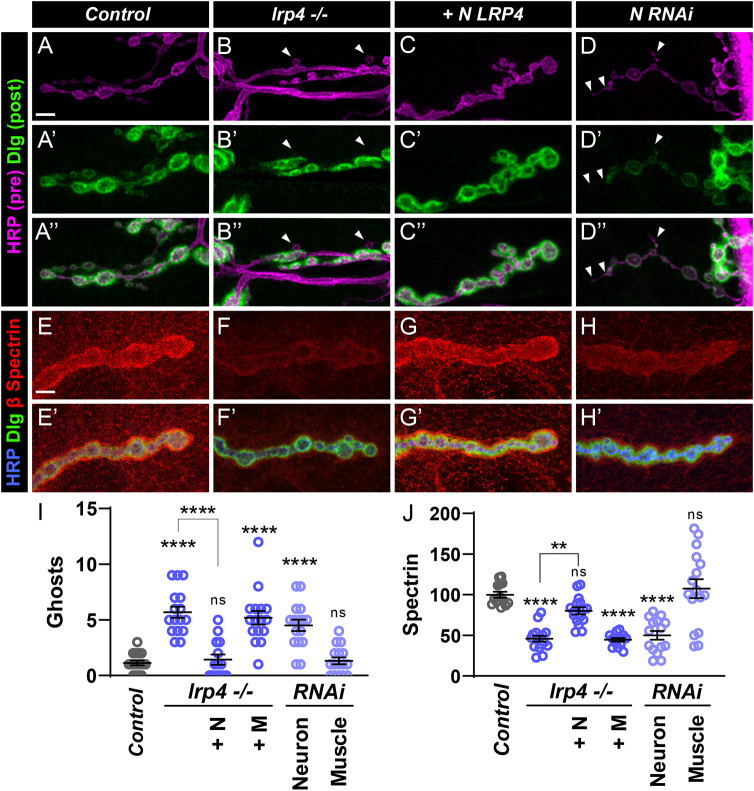
**Motoneuron LRP4 is required for synapse maturation.** (A-D″) Representative confocal images of NMJs from control (A-A″), *lrp4* mutant (B-B″), *lrp4* mutant expressing LRP4 in motoneurons (C-C″) and *lrp4* motoneuron RNAi (D-D″) larvae stained with antibodies to Dlg (green) and HRP (magenta). Arrowheads indicate ghost boutons that lack Dlg staining. Scale bar: 5 µm. (E-H′) Representative confocal images of NMJs from control (E,E′), *lrp4* mutant (F,F′), *lrp4* mutant expressing LRP4 in motoneurons (G,G′) and *lrp4* motoneuron RNAi (H,H′) larvae stained with antibodies to β-Spectrin (red), Dlg (green) and HRP (blue). Scale bar: 5 µm. (I) Quantification of ghost boutons. +N, expression in motoneurons; +M, expression in muscle. (J) Quantification of spectrin fluorescence intensity levels (A.U.). +N, expression in motoneurons; +M=, expression in muscle. For all experiments, data are mean±s.e.m. **P<0.01, ****P<0.0001. ns, not significant. Significance was calculated using one-way ANOVA, followed by Tukey's test for multiple comparisons. *n*≥14 NMJs, *n*≥7 larvae.

We first examined whether LRP4 promotes synaptic maturation by examining pre- and postsynaptic markers, and quantifying the number of ghost boutons at *lrp4* mutant NMJs (Fig. 4A,B). Compared with controls, loss of *lrp4* increased ghost boutons fourfold (Fig. 4A,B,I). The maturation defect can be rescued through expression of LRP4 in motoneurons (Fig. 4C), but not in muscle (Fig. S7B, Fig. 4I), suggesting that presynaptic LRP4 is necessary for synaptic maturation and postsynaptic protein recruitment. Presynaptic *lrp4* RNAi in motoneurons (Fig. 4D), but not in muscles (Fig. S7C, Fig. 4I), also significantly increased ghost bouton occurrence. We next examined postsynaptic β-spectrin staining surrounding boutons (Fig. 4E,F). Loss of *lrp4* reduced spectrin fluorescence intensity by 50% compared with controls (Fig. 4E,F,J). This decrease can be rescued by expression of LRP4 in motoneurons (Fig. 4G), but not in muscle (Fig. S7E, Fig. 4J). Knockdown of *lrp4* in motoneurons (Fig. 4D), but again not in muscles (Fig. S7F, Fig. 4J), also similarly reduces spectrin levels. In all, our data indicate that presynaptic LRP4 ensures normal postsynaptic maturation and cytoskeletal organization, suggesting presynaptic LRP4 may be broadly required for cytoskeletal organization on both sides of the synapse.

### Loss of *lrp4* perturbs synaptic ultrastructure

**Fig. 5.**
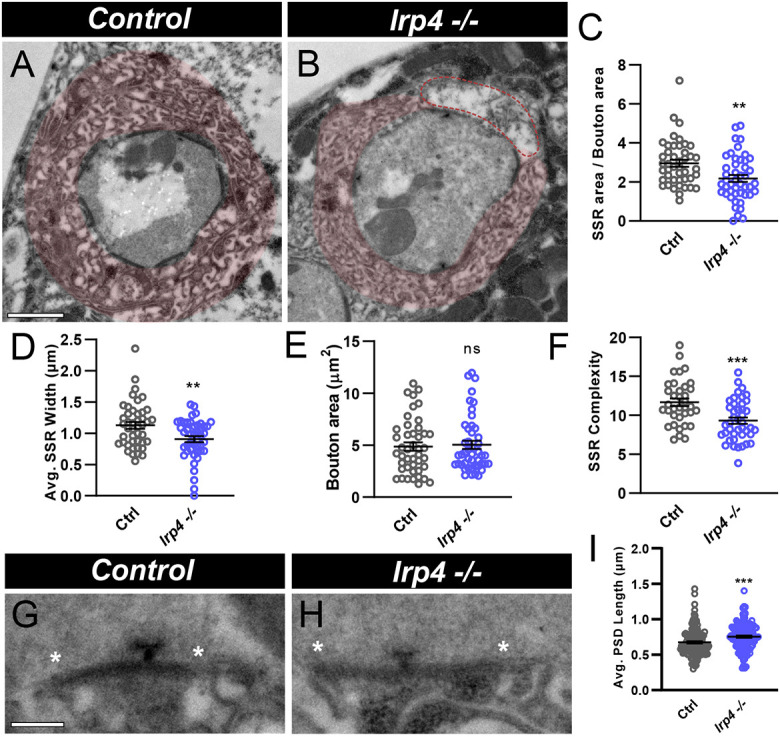
**Loss of *lrp4* leads to ultrastructural defects in membrane complexity.** (A,B) Representative electron micrographs of boutons from control (A) and *lrp4* mutant (B) larvae with SSR false-colored in red. Dotted lines in B indicate a region of disorganized SSR. Scale bar: 1 µm. Asterisks indicate the boundaries of PSDs. Scale bar: 200 nm. (C) Quantification of SSR area normalized to bouton area. (D) Quantification of average SSR width. (E) Quantification of bouton area. (F) Quantification of average membrane crossings/SSR complexity. (G,H) Representative electron micrographs of PSDs from control (G) and *lrp4* mutant (H) larvae. (I) Quantification of average PSD length. For all experiments, data are mean±s.e.m. **P<0.01, ***P<0.001. ns, not significant. Significance was determined using a two-tailed Student's *t*-test. *n*≥44 boutons, *n*≥3 larvae.

At the ultrastructural level, boutons appear as discrete structures with synaptic vesicles clustered around presynaptic active zones called T-bars (Feeney et al., 1998; Jia et al., 1993), and are surrounded by a membranous folded structure: the subsynaptic reticulum (SSR). The SSR consists of postsynaptic membranes that contain neurotransmitter receptor, scaffolding and signaling proteins (Prokop, 2006). We examined *lrp4* mutants using ultrastructural analysis because structural defects in active zones and the SSR that are evident via electron microscopy (EM) are often not observed at the light level. Compared with wild type (Fig. 5A), synaptic boutons in *lrp4* mutants (Fig. 5B) have a 26% reduction in SSR area (Fig. 5C) and a 19% reduction in SSR width (Fig. 5D), although bouton area was unchanged (Fig. 5E). The remaining SSR present in *lrp4* mutants has significantly reduced membrane infolding (Fig. 5F), consistent with impaired maturation (Mosca et al., 2012; Mosca and Schwarz, 2010). SSR complexity defects likely correspond to reduced spectrin (Fig. 4), as spectrin coincides with the SSR and is required for its formation (Pielage et al., 2006). We also observed regions of discontinuous or disorganized SSR in *lrp4* mutants that were never observed in wild-type boutons (Fig. 5A,B). Taken together, these defects in the SSR suggest that LRP4 is required for the organization and normal biogenesis of postsynaptic membranes.

*lrp4* mutants (Fig. 5H) also displayed ultrastructural defects at the level of individual release sites when compared with control (Fig. 5G). We quantified presynaptic parameters in our EM dataset and found that, although there were no changes evident in bouton perimeter or T-bar number/length (Fig. S8A-C), there was a significant increase in postsynaptic density (PSD) length in *lrp4* mutants compared with wild type (Fig. 5I). The postsynaptic spectrin cytoskeleton is also required for proper postsynaptic density size – loss of spectrin results in longer PSDs (Pielage et al., 2006), consistent with our findings. The data demonstrate an important role for LRP4 in the biogenesis of synaptic SSR membranes and in development of the postsynaptic density, consistent with defects in active zone organization and synaptic maturation.

### The SR protein kinase SRPK79D functions in the same genetic pathway as LRP4

Our data thus far highlight a role for presynaptic LRP4 in diverse aspects of synaptic organization beyond previous understanding. We next sought to ascertain the downstream molecular effectors by which presynaptic LRP4 promotes synaptic development. In mammals, LRP4 often acts upstream of MuSK (Kim et al., 2008; Zhang et al., 2008) but as *Drosophila* lack a MuSK homologue, this kinase cannot function downstream of fly LRP4. Previous work in the *Drosophila* CNS implicated a different kinase – the SR-protein kinase SRPK79D – downstream of LRP4 (Mosca et al., 2017). SR-family kinases were originally identified in mRNA splicing but were more recently found to function throughout the cell (Giannakouros et al., 2011), including in the nervous system (Arancibia et al., 2019; Bustos et al., 2020; Chan and Ye, 2013). At the *Drosophila* NMJ, SRPK79D localizes presynaptically (Fig. S9A) at active zones (Johnson et al., 2009) and regulates active zone assembly via Brp phosphorylation (Driller et al., 2019; Johnson et al., 2009; Nieratschker et al., 2009), but how SRPK79D is regulated at NMJ synapses and what molecules function upstream remain unknown.

**Fig. 6.**
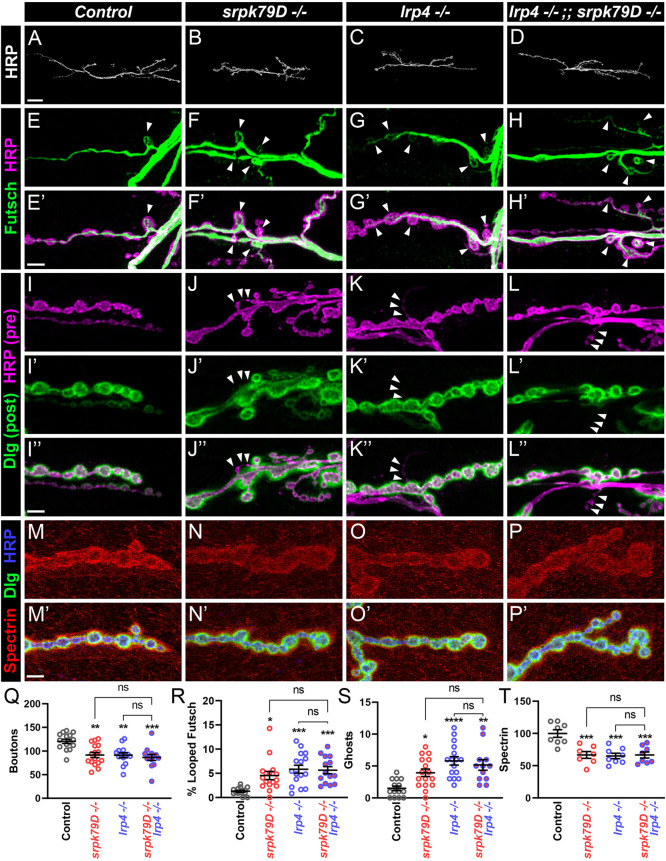
**Loss of *srpk79D* phenocopies the loss of *lrp4* and functions in the same genetic pathway as *lrp4*.** (A-D) Representative confocal images of NMJs from control (A), *srpk79D* mutant (B), *lrp4* mutant (C) and *lrp4, srpk79D* double mutant (D) larvae stained with antibodies to HRP. Scale bar: 25 µm. (E-H′) Representative confocal images of NMJs from control (E,E′), *srpk79D* mutant (F,F′), *lrp4* mutant (G,G′) and *lrp4, srpk79D* double mutant (H,H′) larvae stained with antibodies to Futsch (green) and HRP (magenta). Arrowheads indicate Futsch loops. Scale bar: 5 µm. (I-L″) Representative confocal images of NMJs from control (I-I″), *srpk79D* mutant (J-J″), *lrp4* mutant (K-K″) and *lrp4, srpk79D* double mutant (L-L″) larvae stained with antibodies to Dlg (green) and HRP (magenta). Arrowheads indicate ghost boutons. Scale bar: 5 µm. (M-P′) Representative confocal images of NMJs from control (M,M′), *srpk79D* mutant (N,N′), *lrp4* mutant (O,O′) and *lrp4, srpk79D* double mutant (P,P′) larvae stained with antibodies to β-Spectrin (red), Dlg (green) and HRP (blue). Scale bar: 5 µm. (Q) Quantification of bouton number from A-D. (R) Quantification of percentage of boutons containing Futsch loops from E-H′. (S) Quantification of ghost boutons per NMJ from I-L″. (T) Quantification of spectrin fluorescence intensity levels (A.U.) from M-P′. For all experiments, data are mean±s.e.m. *P<0.05, **P<0.01, ***P<0.001, ****P<0.0001. ns, not significant. Significance was calculated using one-way ANOVA, followed by Tukey's test for multiple comparisons. *n*≥7 NMJs, *n*≥4 larvae.

We hypothesized that SRPK79D acts downstream of LRP4 to regulate synapse development. To test this hypothesis, we first assessed whether perturbation of *srpk79D* (FBgn0025702) affects synapse development and growth. If SRPK79D functions downstream of LRP4 or otherwise in the same genetic pathway, we expect *srpk79D* loss to phenocopy the loss of *lrp4*. To disrupt SRPK79D function, we used a previously validated *srpk79D* mutant, *srpk79D^atc^* (Johnson et al., 2009). Loss of *srpk79D* significantly decreased bouton number (Fig. 6A,B,Q) by 24%, similar to *lrp4* mutants, indicating a role for SRPK79D in NMJ growth. We also observed similar alterations to *lrp4* mutants in the microtubule cytoskeleton of *srpk79D* mutants. *srpk79D* mutants showed a greater than threefold increase in the percent of boutons containing Futsch loops (Fig. 6E,F,R), indicating a shared phenotype with *lrp4* and demonstrating a role for SRPK79D in the organization of the microtubule cytoskeleton. Perturbations of *srpk79D* also showed defects in active zone organization and in synaptic maturation, similar to *lrp4* mutants. Loss of *srpk79D* resulted in more unapposed Brp or GluRIIC puncta at active zones (Fig. S10A-C), a fourfold increase in the number of ghost boutons (Fig. 6I,J,S) and a concomitant decrease in postsynaptic spectrin fluorescence (Fig. 6M,N,T). These combined data suggest that, via multiple metrics assaying distinct aspects of development, *srpk79D* mutants phenocopy the *lrp4* mutants.

The data next led us to determine whether *lrp4* and *srpk79D* interact genetically and function together in the same pathway or in parallel pathways to instruct synapse development. We assessed a potential interaction using a double mutant approach; we reasoned that if LRP4 and SRPK79D function together, disruption of both would lead to similar phenotypes as mutation of either gene (i.e. it would not enhance the phenotype). Conversely, if they functioned independently in parallel pathways, we would expect loss of both genes to enhance each other, resulting in a more severe phenotype. Importantly, we observed no significant differences in the double *lrp4*; *srpk79D* mutant compared with either single mutant in bouton number (Fig. 6B-D,Q), microtubule organization (Fig. 6F-H,R), ghost bouton number (Fig. 6J-L,S) or spectrin fluorescence (Fig. 6N-P,T). Our findings thus support a mechanism whereby LRP4 and SRPK79D likely function together in the same genetic pathway, and not in parallel pathways.

### SRPK79D functions downstream of LRP4 to instruct synapse development

**Fig. 7.**
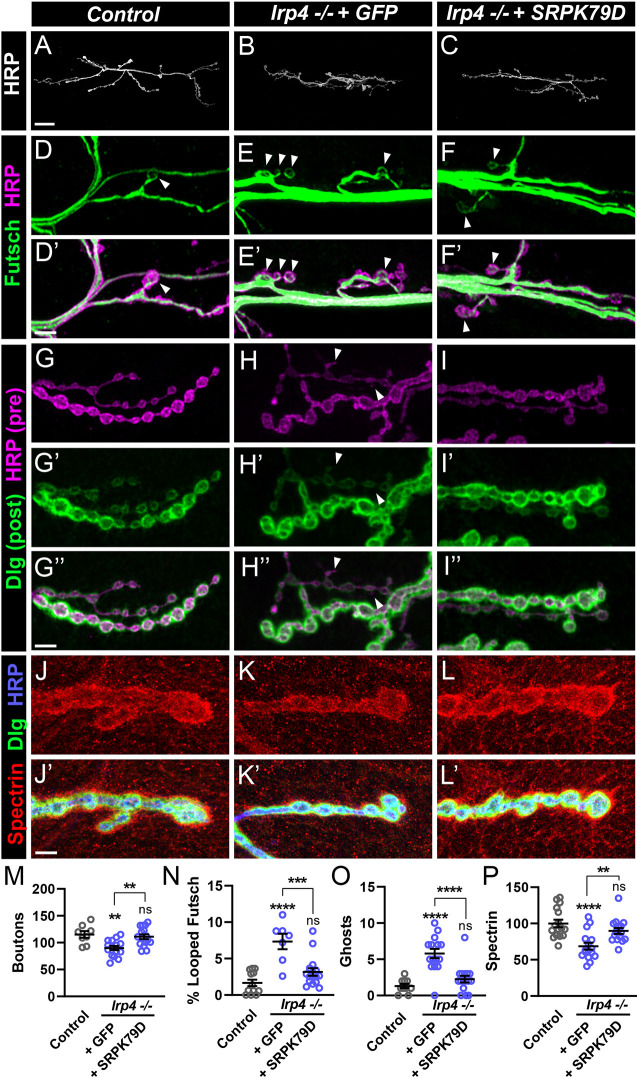
**Overexpression of SRPK79D suppresses *lrp4* mutant growth and maturation phenotypes.** (A-C) Representative confocal images from control (A) and *lrp4* mutant NMJs expressing GFP (B) or SRPK79D (C) in motoneurons larvae stained with antibodies to HRP. Scale bar: 25 µm. (D-F′) Representative confocal images from control (D,D′), and *lrp4* mutant NMJs expressing GFP (E,E′) or SRPK79D (F,F′) in motoneurons stained with antibodies to Futsch (green) and HRP (magenta). Arrowheads indicate Futsch loops. Scale bar: 5 µm. (G-I″) Representative confocal images from control (G-G″) and *lrp4* mutant NMJs expressing GFP (H-H″) or SRPK79D (I-I″) in motoneurons stained with antibodies to Dlg (green) and HRP (magenta). Arrowheads indicate ghost boutons. Scale bar: 5 µm. (J-L′) Representative confocal images from control (J,J′) and *lrp4* mutant NMJs expressing GFP (K,K′) or SRPK79D (L,L′) in motoneurons stained with antibodies to β-Spectrin (red), Dlg (green) and HRP (blue). Scale bar: 5 µm. (M) Quantification of bouton number from A-C. (N) Quantification of the percentage of boutons containing Futsch loops from D-F′. (O) Quantification of ghost boutons per NMJ from G-I″. (P) Quantification of spectrin fluorescence intensity levels (A.U.) from J-L′. For all experiments, data are mean±s.e.m. **P<0.01, ***P<0.001, ****P<0.0001. ns, not significant. Significance was calculated using one-way ANOVA, followed by Tukey's test for multiple comparisons. *n*≥7 NMJs, *n*≥4 larvae.

**Fig. 8.**
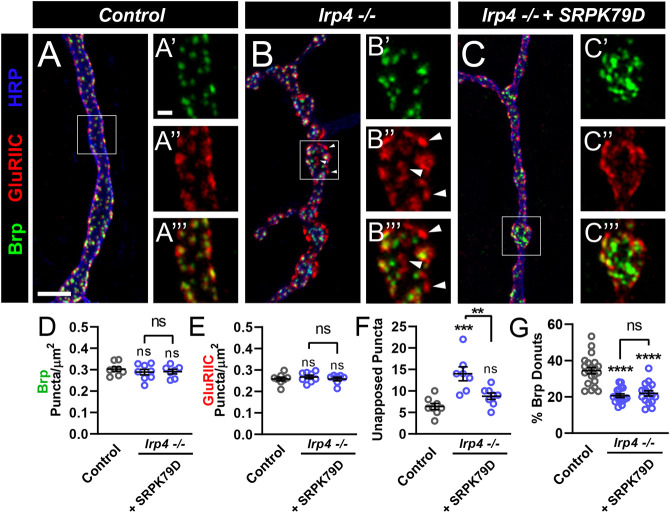
**Overexpression of SRPK79D suppresses *lrp4* mutant apposition defects.** (A-C‴) Representative confocal images from control (A-A‴), *lrp4* mutant (B-B‴) and *lrp4* mutant NMJs expressing SRPK79D in motoneurons (C-C‴) stained with antibodies to Brp (green), GluRIIC (red) and HRP (blue). Arrowheads indicate unapposed puncta. The outlined areas are shown at higher magnification on the right. Scale bars: 5 µm in A-C; 2 µm in A-A‴,B-B‴,C-C‴. (D) Quantification of Brp puncta density from A-C‴. (E) Quantification of GluRIIC density from A-C‴. (F) Quantification of unapposed puncta per NMJ from A-C‴. (G) Quantification of percent of donut-shaped Brp puncta. For all experiments, data are mean±s.e.m. **P<0.01, ***P<0.001, ****P<0.0001. ns, not significant. Significance was calculated using one-way ANOVA, followed by Tukey's test for multiple comparisons. (A-F) *n*≥7 NMJs, *n*=4 larvae. (G) *n*≥16 boutons, *n*=4 larvae.

We finally sought to determine the epistatic relationship between *srpk79D* and *lrp4*; given the association of LRP4 with the synaptic membrane and the synaptic localization of SRPK79D (Johnson et al., 2009), we reasoned that SRPK79D was most likely to function downstream of LRP4 (Mosca et al., 2017). If SRPK79D functions downstream of LRP4, overexpressing SRPK79D in the *lrp4* mutant background should be sufficient to suppress the *lrp4* mutant phenotypes (and not vice versa)*.* Given that our data indicate that LRP4 functions presynaptically, SRPK79D should similarly function in motoneurons. To test this hypothesis, we expressed a Venus-tagged SRPK79D in the motoneurons of *lrp4* mutants and compared these with *lrp4* mutants expressing a control GFP transgene in motoneurons (Fig. 7). To quantify NMJ growth, we counted synaptic boutons (Fig. 7A-C) and observed suppression of the decreased bouton number phenotype after SRPK79D overexpression (Fig. 7M). To assess microtubule organization, we assessed boutons containing looped Futsch (Fig. 7D-F), and again observed suppression of the *lrp4* mutant phenotype (Fig. 7N). As metrics of synapse maturation, we assessed ghost boutons (Fig. 7G-I,O) and spectrin fluorescence intensity (Fig. 7J-L,P); in both cases, we observed suppression of *lrp4* mutant phenotypes after motoneuron SRPK79D overexpression. Finally, we assessed active zone and glutamate receptor density and apposition (Fig. 8A-E) and found that SRPK79D expression was sufficient to suppress the active zone and glutamate receptor apposition phenotype observed after loss of *lrp4* (Fig. 8F). Interestingly, SRPK79D overexpression did not suppress the active zone organization defects observed after loss of *lrp4* (Fig. 8G), suggesting an SRPK79D-independent role for LRP4 in the biogenesis of individual active zones. Importantly, overexpression of SRPK79D alone in otherwise wild-type motoneurons shows no significant changes in synaptic organization compared with control (Fig. S11). Further consistent with our hypothesis, overexpression of LRP4 in *srpk79D* mutants failed to suppress any defects in synapse organization, supporting a role for LRP4 upstream of SRPK79D (Fig. S12). These data indicate that SRPK79D overexpression is sufficient to suppress multiple *lrp4* mutant phenotypes in synaptogenesis and suggest that SRPK79D functions downstream of presynaptic LRP4 in motoneurons to regulate diverse developmental processes in a core neuronal signaling pathway that promotes NMJ development.

## DISCUSSION

The coordination of intricate cellular processes during synapse development is critical to forming robust and lasting connections. Here, we find that the receptor LRP4 acts presynaptically at peripheral *Drosophila* synapses as a master organizer of synapse development, including growth, cytoskeletal structure, active zone organization and maturation. We show that the SR-protein kinase SRPK79D functions downstream of LRP4 at the NMJ to promote development. These findings begin to answer critical open questions in LRP4-related and synaptic biology. Although postsynaptic functions of LRP4 are well documented (DePew and Mosca, 2021), how LRP4 may act presynaptically in any system is notably less well understood. First, we highlight that LRP4 functions in presynaptic motoneurons at neuromuscular synapses, which has previously been unclear and controversial. Second, we reveal that LRP4 influences a range of synaptic developmental processes leading to formation of reliable connections. Third, we identify previously unreported roles for LRP4 in regulating the synaptic cytoskeleton and synaptic maturation. Finally, we identify a shared downstream mechanism for LRP4 in the SRPK79D kinase that unites the roles of LRP4 as a master regulator of synaptic organization. These findings not only contribute to our understanding of mechanisms of synapse development in *Drosophila* but can also inform understanding of LRP4 at other synapses. Given the mechanistic conservation between central and peripheral synapses in *Drosophila* (Mosca et al., 2017), our work may provide unique insight on potential mechanisms in the mammalian CNS where the complete function of LRP4 remains elusive (Gomez et al., 2014; Handara et al., 2019; Karakatsani et al., 2017).

### LRP4 as a presynaptic organizer of active zones

Pioneering work identified a crucial role for postsynaptic LRP4 in mammalian NMJ synaptogenesis as the co-receptor for the synaptogenic ligand Agrin (Kim et al., 2008; Weatherbee et al., 2006; Zhang et al., 2008). LRP4 also influences presynaptic differentiation, as LRP4 is likely cleaved and acts retrogradely to instruct motoneuron development (Yumoto et al., 2012). In this last case, the source of LRP4 is still postsynaptic but functions trans-synaptically. Other presynaptic roles for LRP4 at the NMJ remained controversial. Some genetic evidence suggests LRP4 acts presynaptically in synaptic maintenance (Wu et al., 2012), but this is not well understood and the image of LRP4 has remained solely as a postsynaptic protein. Recent work has begun to suggest a duality for LRP4, as both a presynaptic and a postsynaptic factor. In the central nervous system, evidence highlights a role for LRP4 in regulating synapse formation (Gomez et al., 2014; Handara et al., 2019; Karakatsani et al., 2017; Mosca et al., 2017). LRP4 acts presynaptically at *Drosophila* olfactory synapses to regulate active zones, although there is no mechanistic insight into other developmental processes and no postsynaptic role has been identified (Mosca et al., 2017). At mammalian central synapses, LRP4 is enriched at pre- and postsynaptic membranes, and its perturbation leads to both pre- and postsynaptic defects (Karakatsani et al., 2017); however, the underlying mechanisms are also not understood. In the mammalian brain, most evidence suggests LRP4 may act postsynaptically or in glia to regulate synapse biology; however, this is largely unknown (Sun et al., 2016; Tian et al., 2006; Zhang et al., 2020). Using the fly NMJ, we sought to address potential presynaptic roles of LRP4 with cell type-specific precision. We find that presynaptic LRP4 controls active zone apposition and structure, synaptic function, microtubule organization, and bouton growth. Intriguingly, we find that presynaptic LRP4 also influences postsynaptic maturation, highlighting a trans-synaptic function for presynaptic LRP4 in regulating postsynaptic protein recruitment. How these roles may be conserved at mammalian central synapses, where the precise function of LRP4 is not fully understood, remains a fascinating topic for future study and offers critical insight into presynaptic functions of LRP4.

### LRP4 as a cytoskeletal regulator during growth and maturation

The importance of cytoskeletal dynamics in regulating synaptic biology cannot be understated (Goellner and Aberle, 2012) but how cytoskeletal activity is integrated with, and responds to, signals from the cell surface is less well understood. Our discovery of a role for presynaptic LRP4 in cytoskeletal organization provides a unique opportunity to associate synaptic organizers at the membrane with the cytoskeleton. Microtubule dynamics greatly influence synaptic growth in multiple systems, and in *Drosophila*, perturbation of microtubule regulators can dramatically alter bouton number (Chou et al., 2020a). Consistent with this idea, we found that the absence of presynaptic LRP4 altered cytoskeletal stability, measured by an increase in looped microtubule structures. Improper microtubule stabilization can result in reduced plasticity and may correlate with decreased growth (Chou et al., 2020a) and branching (Parato and Bartolini, 2021). We propose that presynaptic microtubules are improperly stabilized in the absence of LRP4, leading to less synaptic growth. As the microtubule cytoskeleton also influences active zone organization (Koch et al., 2008; Lepicard et al., 2014), the defects in active zone organization we observe may also result from LRP4 influence on microtubule organization. Interestingly, Futsch/MAP1B functions in part as an intermediate between active zones and microtubules, potentially contributing to active zone stability (Lepicard et al., 2014). As a result, we suggest that altered microtubule stability may signify the primary defect in *lrp4* mutants from which alterations in bouton growth and active zone organization stem, with LRP4 serving as a nexus between the active zone machinery and the cytoskeleton. Whether such a role could be mechanistically conserved in vertebrate neurons remains unknown. Mammalian LRP4 in CNS neurons is also essential for dendritic arborization and morphogenesis (Handara et al., 2019; Karakatsani et al., 2017), raising the tantalizing possibility that LRP4 may also function in the mammalian CNS to instruct microtubule organization. Given the contribution of microtubule dynamics to neurodevelopmental disease (Lasser et al., 2018), this insight may inform our understanding of synapse development in both health and disease.

Cytoskeletal organization also promotes synaptic maturation: postsynaptic maturation recruits components such as spectrin to nascent boutons, producing functional connections (Ataman et al., 2006; Li et al., 2007; Mathew et al., 2005; Mosca et al., 2012; Mosca and Schwarz, 2010; Owald et al., 2010; Packard et al., 2002, 2015; Restrepo et al., 2022; Speese et al., 2012). Presynaptic LRP4 is important for postsynaptic spectrin recruitment and SSR organization, indicating, first, a trans-synaptic role for LRP4 signaling and, second, a deeper role in cytoskeleton organization. The picture that emerges is that presynaptic LRP4 is required to regulate the cytoskeletal elements that promote multiple stages of development. In *Drosophila,* many pathways influence synapse maturation, including Wnt signaling (Chou et al., 2020a) that remodels the microtubule cytoskeleton (Gögel et al., 2006; Packard et al., 2002) via LRP5/6 (*Drosophila* Arrow) at the NMJ (Miech et al., 2008; Wehrli et al., 2000). Although these pathways share similarities with LRP4, there are notable differences, suggesting that LRP4 does not simply function to promote Wnt signaling (as loss of *lrp4* does not phenocopy loss of *wg*). Thus, LRP4 likely acts via some (if not all) Wnt-independent pathways. However, this does not rule out an involvement, perhaps through a more complicated regulatory mechanism, as in mammalian early forebrain development (Ahn et al., 2013, 2017; Choi et al., 2009; Geng et al., 2023).

### SRPK79D functions with LRP4 to instruct development

SR-protein specific kinases (SRPKs) were initially discovered to phosphorylate SR proteins and promote the subsequent nuclear import of mRNA splicing factors (Zhou and Fu, 2013). More recent work, however, has demonstrated numerous roles in neuronal development and disease (Bustos et al., 2020; Chan and Ye, 2013; Giannakouros et al., 2011; Hogg and Findlay, 2023). One *Drosophila* SRPK homologue, SRPK79D, has emerged as an important player in NMJ active zone biology (Driller et al., 2019; Johnson et al., 2009; Nieratschker et al., 2009). To date, however, no clear upstream interactor has been identified for SRPK79D at the NMJ, leaving its regulatory mechanisms undiscovered. We found that *srpk79D* and *lrp4* act in the same genetic pathway with LRP4 upstream of SRPK79D for most aspects of development. But how might LRP4 and SRPK79D promote presynaptic differentiation? We posit a model wherein LRP4 signals from the cell membrane, likely in response to an unknown ligand, to enable SRPK79D–mediated cytoskeletal regulation. This reorganization of the cytoskeleton may then directly influence further downstream developmental events, or SRPK79D may act on multiple downstream targets to influence diverse cellular processes. Previous work demonstrates a parsimonious role for SRPK79D in active zone assembly and organization (Driller et al., 2019). Thus, the LRP4 and SRPK79D pathway may either directly or indirectly instruct multiple downstream events to influence presynaptic development. Finally, we identify an LRP4-dependent/SRPK79D-independent mechanism that regulates the shape of individual active zones, suggesting potential further diversity in developmental pathways.

Beyond active zone assembly, we find that LRP4 and SRPK79D are required for cytoskeletal organization but the underlying mechanism remains unknown. One possibility arises from mammalian work demonstrating a role for SRPK2 in neuronal microtubule polymerization via phosphorylation of the microtubule-associated protein Tau (Hong et al., 2012). Like Futsch, Tau is involved in microtubule stabilization (Götz et al., 2006; Lee and Rook, 1992), suggesting SRPKs may function neuronally to regulate the delicate organization of microtubules via phosphorylation. Such a mechanism for SRPK79D would parallel that of another kinase, Shaggy (GSK3β), which influences growth and microtubule stability by phosphorylating Futsch (Franco et al., 2004; Gögel et al., 2006). Although loss of Shaggy increases microtubule loops, it also increases bouton number (Franco et al., 2004), in contrast to disrupting *lrp4* or *srpk79D*. This suggests that multiple concurrent mechanisms regulate the complex balance of microtubule dynamics. Understanding the intersection of multiple mechanisms involving microtubule stability and phosphorylation targets will provide insight into the complex cytoskeletal dynamics that underlie synapse development.

### Limitations of this study

Our analyses of LRP4 demonstrate its diverse conserved roles in synapse biology (DePew and Mosca, 2021). Likewise, in mammals, loss of LRP4 at central synapses results in decreased synapse density (Handara et al., 2019; Karakatsani et al., 2017). Although we observed no changes in active zone density at the NMJ in *lrp4* mutants, the total number of active zones is likely reduced, as fewer boutons are present, demonstrating a conserved role for LRP4 in promoting synapse number. Furthermore, although we observe a significant decrease in neurotransmitter release in *lrp4* mutants, defects in active zone apposition appear mild. How the organizational defects we observe in active zone structure, cytoskeletal organization or synaptic apposition can account for this functional deficit remains unknown. One possibility is that LRP4 functions in additional aspects of presynaptic development. Finally, recent work in the mammalian CNS presents another interesting possibility. There, astrocytic LRP4 is implicated in the modulation of glutamate release (Sun et al., 2016). This is unlikely to play a role at the *Drosophila* NMJ, as LRP4 expression is limited to presynaptic motor neurons, and glial expression of LRP4 does not rescue *lrp4* mutant defects (Fig. S5). However, a glial role for LRP4 in *Drosophila* cannot be conclusively ruled out, perhaps in modulating glutamate release in the CNS, and remains a fascinating topic for future investigation.

## MATERIALS AND METHODS

### *Drosophila* stocks, transgenic strains and genotypes

All controls, stocks and crosses were maintained on cornmeal medium (Archon Scientific, Durham, NC, USA) at 25°C and 60% relative humidity with a 12/12 light/dark cycle in specialized incubators (Darwin Chambers, St Louis, MO, USA). Canton S was used as the control line unless otherwise noted. All mutants and transgenes were maintained over larvally selectable balancer chromosomes to enable identification. The following mutant alleles were used: *lrp4^dalek^* (Mosca et al., 2017) and *srpk79D^atc^* (Johnson et al., 2009). The following UAS transgenes were used: *UAS-lrp4-HA* (Mosca et al., 2017), *UAS-mCD8-GFP* (Lee and Luo, 1999), *UAS-lrp4-*RNAi (108629, Vienna Drosophila Resource Center), *UAS-Dcr2* (Dietzl et al., 2007) and *UAS-venus-SRPK79D-#28* (Johnson et al., 2009). *GMR90B08-GAL4* (referred to as *lrp4-GAL4*) was used to drive expression in cells expressing LRP4 (Pfeiffer et al., 2008). *C155-GAL4* (Lin and Goodman, 1994) was used to drive expression pan-neuronally. *OK6-GAL4* (Aberle et al., 2002) was used to drive expression in motoneurons. *Mhc-GAL4* (Schuster et al., 1996) or *DMef2-GAL4* (Lilly et al., 1995) was used to drive expression in all somatic muscles. *Repo-GAL4* (Lee and Jones, 2005) was used to drive expression in all glia. See Table S1 for genotypes.

### Construction of fly lines

An 3xHA-tag was knocked in to the endogenous *lrp4* locus to enable visualization of endogenous LRP4. We used CRISPR/Cas9 genome editing (Gratz et al., 2015), with WellGenetics (New Taipei City, Taiwan) to make a custom-designed guide RNA and to construct to introduce the 3xHA tag. We chose to tag LRP4 at the C terminus, as a previous attempt generating a transgenic UAS line of LRP4 tagged at its C terminus was successful (Mosca et al., 2017). Four lines were obtained and sequenced to confirm the presence of the 3xHA-tag, and each line was balanced over FM7a. We also used CRISPR to delete the *lrp4*-coding sequence and generate an independent knockout allele *lrp4^Del^* (Gratz et al., 2013). The deletion was confirmed by sequencing.

### Immunocytochemistry

Larvae were dissected and stained as previously described (Mosca and Schwarz, 2010; Restrepo et al., 2022), and raised in population cages (Genesee, no. 59-100) on grape juice plates supplemented with yeast paste. Wandering third instar larvae were dissected in Ca^2+^ -free modified *Drosophila* saline (White et al., 2001). Larval fillets were fixed in 4% paraformaldehyde in 1×PBST for 20 min followed by three 20 min washes in PBST, and a 1 h block in 5% normal goat serum. Samples were incubated in primary antibodies overnight, followed by three 10 min washes in PBST, and incubation in secondary antibodies for 2 h at room temperature. The following primary antibodies were used: mouse anti-Brp (DSHB, mAbnc82, 1:250) (Laissue et al., 1999), mouse anti-Dlg (DSHB, mAb4F3, 1:500) (Parnas et al., 2001), rabbit anti-GluRIIC (1:2500; Marrus et al., 2004), rabbit anti-GluRIIC (1:2000; Sulkowski et al., 2014), mouse anti-Futsch (DSHB, mAb22C10, 1:50) (Roos et al., 2000), rabbit anti-β Spectrin (1:1000; Byers et al., 1989) and rabbit anti-HA RM305 (RevMab Biosciences, 31-1190-00, 1:500). Alexa488- and Alexa647- (Jackson ImmunoResearch, 711-545-152, 711-605-152, 715-545-151 and 115-605-166), and Alexa568- (ThermoFisher, 1793903 and 1832035) conjugated secondary antibodies were used at 1:250. Cy3- or Alexa647-conjugated goat anti-HRP primary antibodies were used at 1:100 (Jackson ImmunoResearch, 123-165-021 and 123-605-021). Samples processed for confocal imaging were mounted in Vectashield Antifade Mounting Medium (Vector Laboratories).

### Confocal imaging and image processing

Confocal *z*-stacks were acquired using a Zeiss LSM880 Laser Scanning Confocal microscope with 40×1.4 NA PlanApo or 63×1.4 NA PlanApo oil objectives.

### STED imaging and deconvolution

For STED imaging, immunocytochemistry protocols were slightly adjusted to improve analysis of active zones (modified from Jetti et al., 2023). After dissection in Ca^2+^ -free modified *Drosophila* saline, samples were fixed in 4% paraformaldehyde for 10 min. After incubation in primary and secondary antibodies, samples were mounted on slides using SlowFade. All STED images were acquired as *z*-stacks using a Leica TCS SP8 STED 3X microscope with a 100× objective. For all STED imaging, mouse anti-Brp (DSHB, mAbnc82, 1:250) primary antibodies were used with Alexa488-conjugated secondary antibodies. To deconvolve STED images, *z*-stacks were first converted to stacked TIFF files using ImageJ and deconvolved using Nikon Elements software. 3D deconvolution was performed using the Landweber algorithm, with maximum 20 iterations. Deconvolved images were maximum intensity projected in ImageJ for analysis. Only type 1b boutons were analyzed.

### Electron microscopy

Wandering third instar larvae were dissected as described above. Samples were fixed in 2.5% PFA, 5% glutaraldehyde and 0.06% picric acid in 0.1 M cacodylate buffer overnight on ice. After fixation, samples were incubated in 2% osmium tetroxide for 1 h on ice. Samples were then dehydrated in an ethanol series, rinsed in propylene oxide and incubated in 50% propylene oxide/50% resin overnight. Samples were then added to fresh resin for 4 h and embedded in an incubator at 65°C for 2 days or until hard. The 6/7 muscle region was identified by taking 1 μm square sections, and bouton regions were located by taking 90 nm sections until muscle tissue was identified. All electron micrographs were acquired using a FEI Tecnai 12 120 keV digital TEM, fitted with a bottom-mounted AMT BioSprint 12 MPx CCD camera.

### Electrophysiology

Spontaneous and evoked postsynaptic potentials were recorded in voltage clamp mode from muscle 6 in male third-third instar larvae as previously described (Bruckner et al., 2017). Larvae were dissected in Ca^2+^-free hemolymph-like saline (HL3), which was replaced with saline containing 0.6 mM Ca^2+^ for recording. mEJPs were recorded for 1 min and 60 were averaged to obtain mEJP amplitude for each muscle before a stimulus was applied. EJPs were evoked in abdominal segments 3 and 4 by suctioning the cut end of the segmental nerve and applying stimulus at 0.5 Hz with stimulus amplitude adjusted to reliably evoke both Is and Ib nerve inputs. At least 25 consecutive EJPs were recorded for each cell and analyzed in pClamp to obtain mean amplitude. Quantal content was calculated for each cell as mean EJP amplitude divided by mean mEJP amplitude.

### Quantification of NMJ synaptic parameters

All NMJs were quantified from muscles 6/7 or muscle 4 on both the left and right sides, and comparisons were made only within larval segments. All phenotypes were also observed at other synapses, regardless of muscle fiber or segment. Bouton number was counted in NMJs of muscles 6/7 by hand at segment A3, unless otherwise noted. Futsch loops and unbundled Futsch were counted by hand at terminals of muscles 6/7. Ghost boutons were quantified as HRP-positive Dlg-negative membrane protrusions with a visible connection to the NMJ terminal, as previously described (Restrepo et al., 2022). Synaptic spectrin fluorescence intensity was measured in ImageJ by drawing a region of interest surrounding the NMJ. Puncta density was determined in muscle 4 using the ‘Spots’ function in Imaris software (Oxford Instruments), with a spot size of 0.4 for Brp puncta and 0.6 for GluRIIC puncta. Unapposed puncta were then counted by hand as either a Brp punctum lacking a corresponding GluRIIC or a GluRIIC punctum lacking Brp.

Electron micrographs were analyzed using ImageJ. Parameters for ultrastructural analysis were quantified as previously described (Mosca and Schwarz, 2010). SSR, PSD and T-bar analysis was performed using ImageJ on boutons that were at least 1 µm in length and contained an active zone. Bouton area was calculated by tracing the perimeter of the bouton, and SSR area was calculated by tracing the perimeter of the bouton and the entire bouton+SSR, and subtracting the area of the bouton. For SSR width, an arbitrary center point of the bouton was chosen, and eight radii were drawn outward from the center at 45° angle intervals. The width of the SSR was measured at each line and averaged. For SSR complexity, eight radii were drawn outward from the center at 45° angle intervals and the numbers of membranes crossing each line were counted by hand and averaged for each bouton.

Figures were constructed using ZEN 2.3 software (Zeiss), ImageJ (NIH), Adobe Photoshop 2023 and Adobe Illustrator 2023.

### Statistical analysis

Statistical analysis was performed and graphical representations prepared using Prism 9.5.1 (Graphpad Software). Data are expressed as mean±s.e.m. Normality was determined using a D'Agostino-Pearson test. Significance between two groups was determined using an unpaired two-tailed Student's *t*-test. Significance among three or more groups was determined using one-way ANOVA with a Dunnett's post-hoc test to a control group and a Bonferroni post-hoc test among all groups. Multiple comparisons were corrected for using a Tukey's post-hoc test. For single comparisons between non-normally distributed data, a Mann–Whitney *U*-test was used. In each figure, unless otherwise noted, statistical significance is indicated in comparison to control genotypes. Exact *n* values are listed in Table S2.

## Supplementary Material



10.1242/develop.202517_sup1 Supplementary information
