# Supplementary material for: Neuronal LRP4 directs the development, maturation and cytoskeletal organization of Drosophila peripheral synapses
Source: Development. 2024 Jun 3;151(11):dev202517. doi: 10.1242/dev.202517 (PMC11190576; doi:10.1242/dev.202517)
Supplement: Supplementary information [file develop-151-202517-s1.pdf]

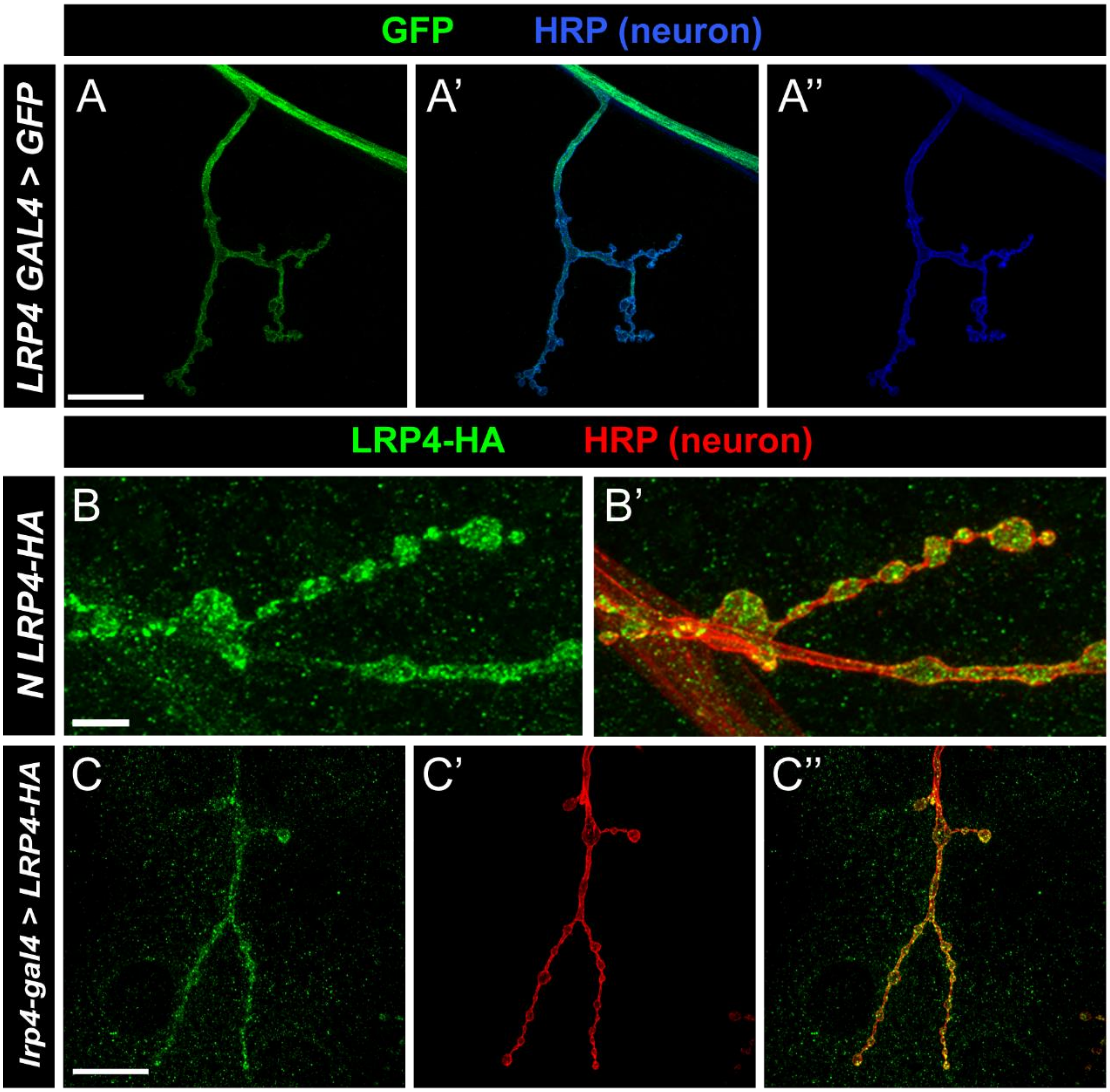

Fig. S1. LRP4 is expressed in motoneurons and localizes in boutons at the NMJ.

(A) Representative confocal image of an NMJ expressing GFP via an *LRP4-GAL4* stained with antibodies to HRP (blue). Scale = 20µm.

(B) Representative confocal image of an NMJ expressing HA-tagged LRP4 pan-neuronally using *C155-GAL4* stained with antibodies to HA (green) and HRP (red). LRP4-HA localizes to boutons. Scale = 5µm.

(C) Representative confocal image of an NMJ expressing HA-tagged LRP4 driven by *LRP4-GAL4* and stained with antibodies to HA (green) and HRP (red). Scale = 20µm.

Development • Supplementary information

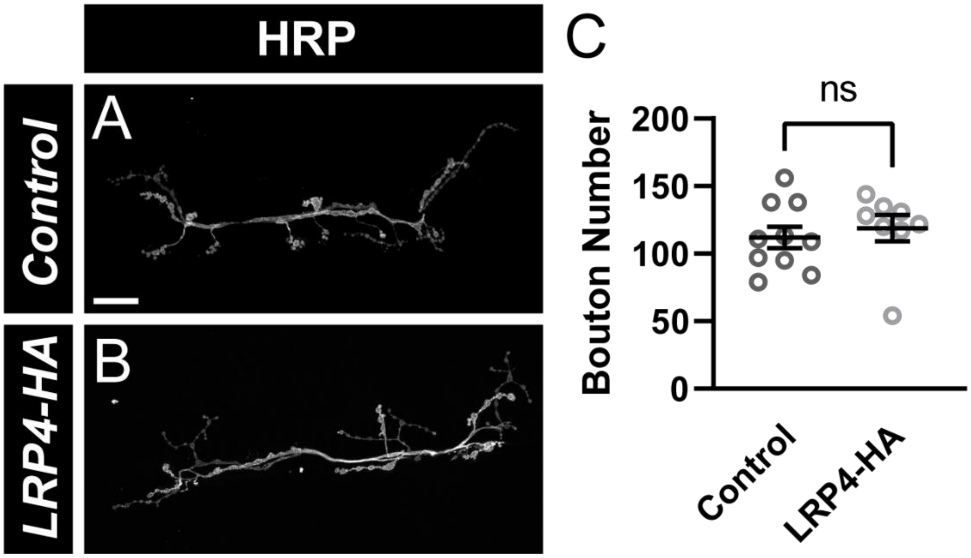

Fig. S2. NMJ morphology appears normal following endogenous tagging of LRP4. (A-B) Representative confocal images from control (A) and endogenous LRP4-HA (B) larvae stained with antibodies to HRP.

(C) Quantification of bouton number.

For all experiments, data are shown as mean ± SEM. *ns* = not significant. Significance was determined using a two-tailed Student's t-test. $n \geq 8$ NMJs, 4 larvae.

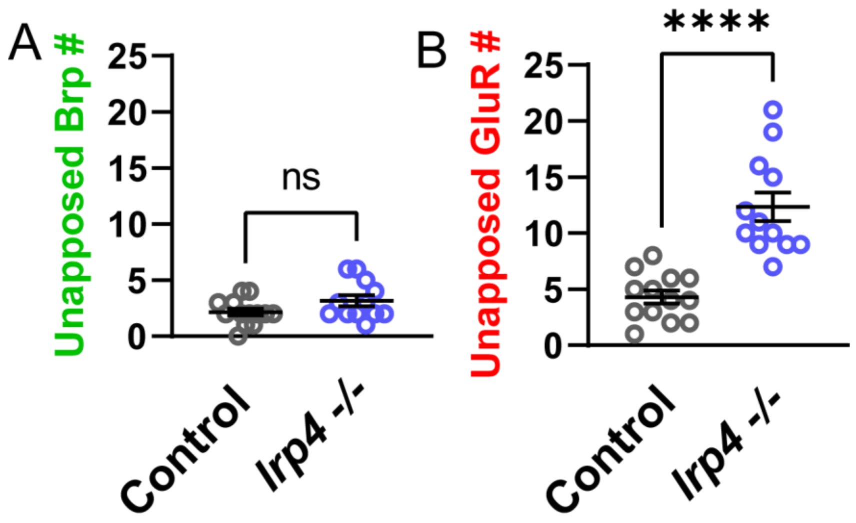

Fig. S3. Loss of *Irp4* results in a significant increase in unapposed GluRIIC puncta.
(A) Quantification of unapposed Brp puncta.

(B) Quantification of unapposed GluRIIC puncta.

For all experiments, data are shown as mean ± SEM. **** $p < 0.001$, *ns* = not significant.

Significance was determined using a two-tailed Student's t-test. $n \geq 12$ NMJs, 6 larvae.

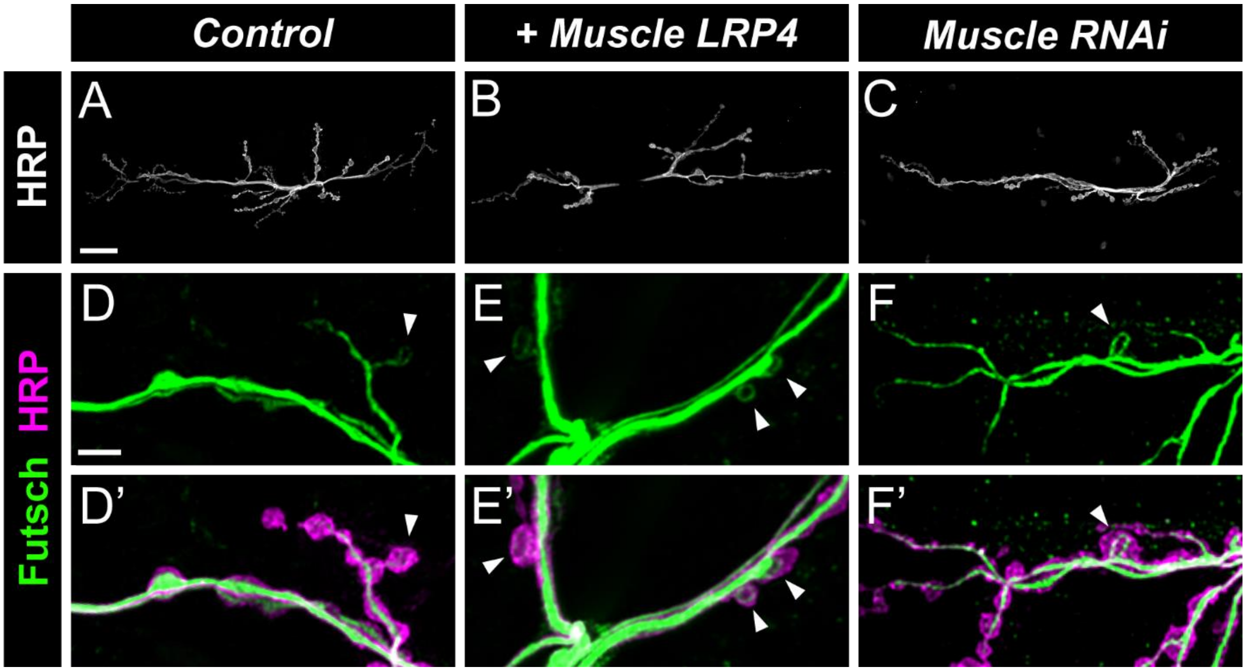

Fig. S4. LRP4 is not required in muscles for synapse growth and microtubule organization.

(A-C) Representative confocal images of NMJs from control (A), *lrp4* mutant expressing LRP4 in muscles (B), and following *lrp4* muscle RNAi (C) larvae stained with antibodies to HRP. Scale = 25μm

(D-F) Representative confocal images of NMJs from from control (D), *lrp4* mutant expressing LRP4 in muscles (E), and *lrp4* muscle RNAi (F) larvae stained with antibodies to Futsch (green) and HRP (magenta). Arrowheads indicate Futsch loops. Scale = 5μm

Development • Supplementary information

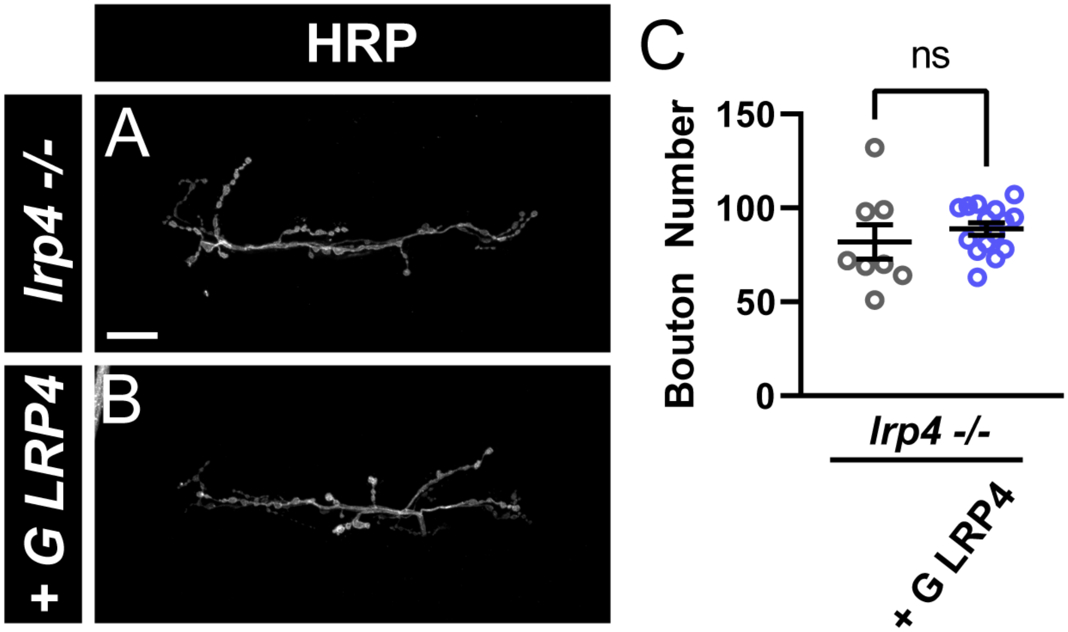

Fig. S5. Glial expression of LRP4 is insufficient to rescue bouton number following loss of *lrp4.*

(A-B) Representative confocal images of NMJs from *lrp4* mutant (A), and *lrp4* mutant expressing LRP4 in glia using *Repo-GAL4* (B) stained with antibodies to HRP. Scale = 25μm

(C) Quantification of bouton number.

For all experiments, data are shown as mean ± SEM. *ns* = not significant. Significance was determined using a two-tailed Student's t-test. $n \geq 8$ NMJs, 4 larvae.

Development • Supplementary information

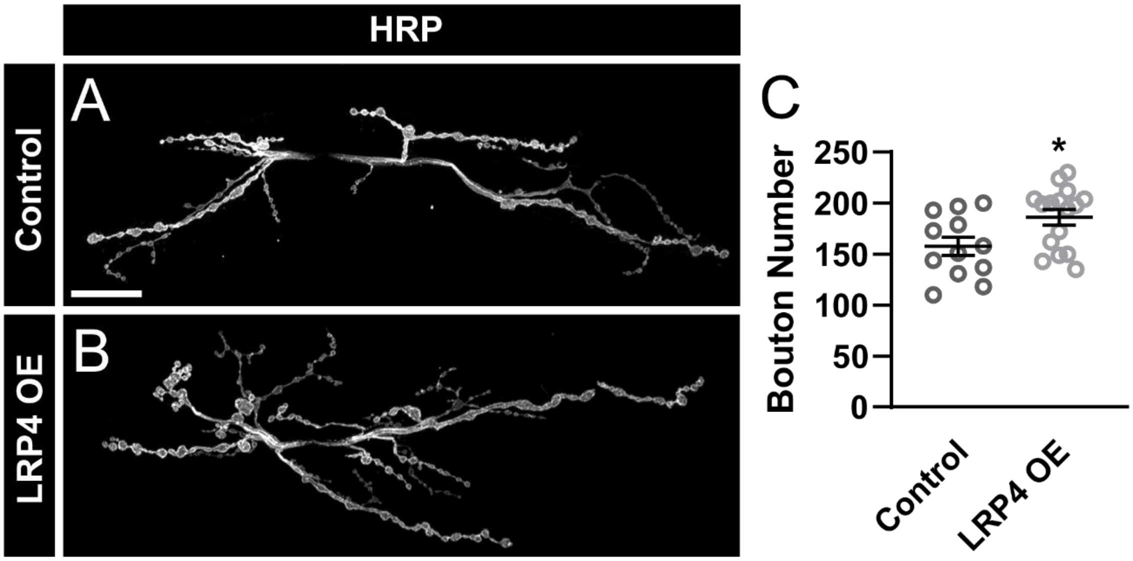

Fig. S6. Overexpression of LRP4 increases bouton number.

(A-B) Representative images of NMJs from control (A) or following overexpression of LRP4 in neurons (B), stained with antibodies to HRP. Scale = 20μm.

(C) Quantification of bouton number.

For all experiments, data are shown as mean ± SEM. * $p < 0.05$. Significance was determined using a two-tailed Student's t-test. $n \geq 12$ NMJs, 7 larvae.

Development • Supplementary information

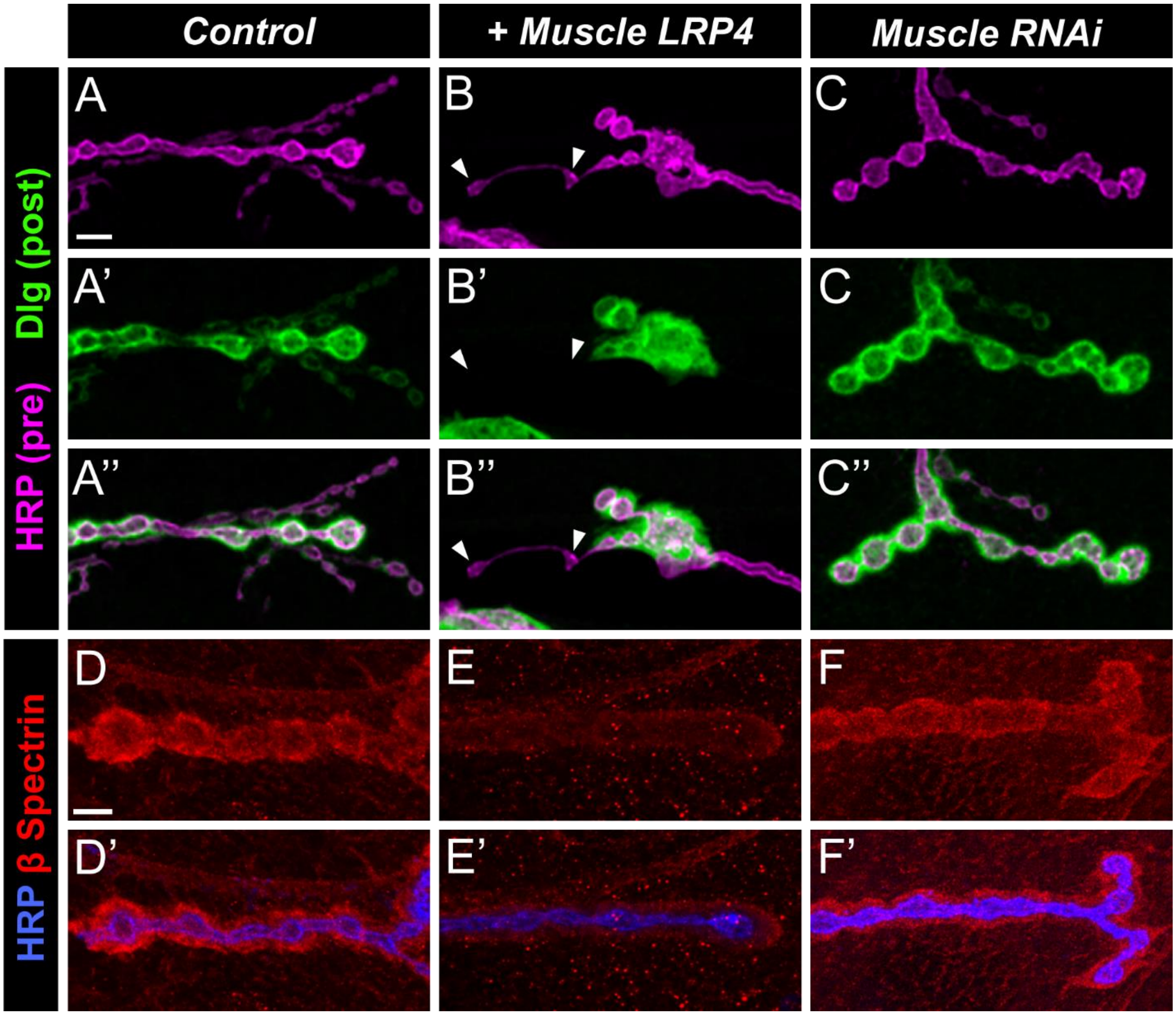

Fig. S7. LRP4 is not required in muscles for synapse maturation.

(A-C) Representative confocal images of NMJs from control (A), *lrp4* mutant expressing LRP4 in muscles (B), and *lrp4* muscle RNAi (C) larvae stained with antibodies to Dlg (green) and HRP (magenta). Arrowheads indicate ghost boutons which lack Dlg staining. Scale = 5μm

(D-F) Representative confocal images of NMJs from from control (D), *lrp4* mutant expressing LRP4 in muscles (E), and *lrp4* muscle RNAi (F) larvae stained with antibodies to β Spectrin (red), Dlg (green), and HRP (blue). Scale = 5μm

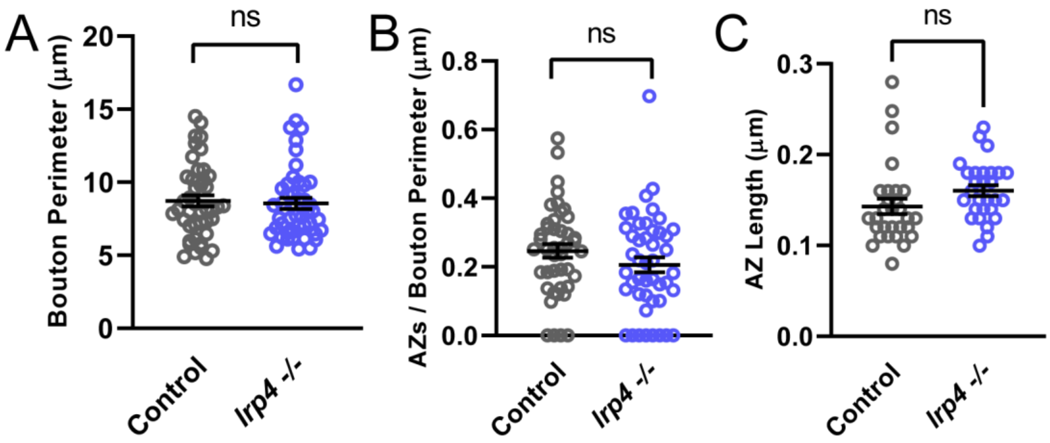

Fig. S8. Loss of *Irp4* does not affect bouton perimeter or active zone number.

(A) Quantification of bouton perimeter.

(B) Quantification of AZs / bouton perimeter.

(C) Quantification of the length of the tabletop of the active zone T-bar.

For all experiments, data are shown as mean ± SEM. *ns* = not significant. Significance was determined using a two-tailed Student's t-test. $n \geq 44$ boutons, 3 larvae.

Development • Supplementary information

Supplemental Figure 9

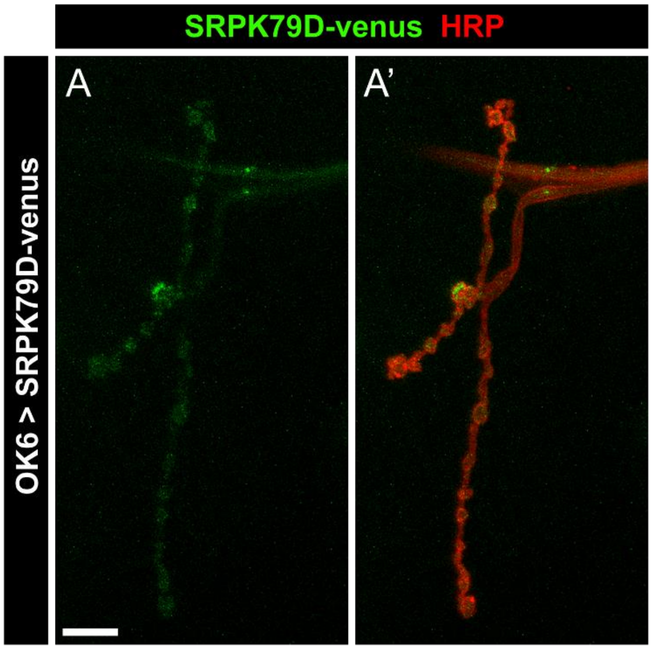

Fig. S9. Tagged-SRPK79D localizes presynaptically.

(A) Representative confocal image of an NMJ expressing venus-tagged SRPK79D (green) in motoneurons using *OK6-GAL4* and stained with antibodies to HRP (red). Scale = 10μm.

Development • Supplementary information

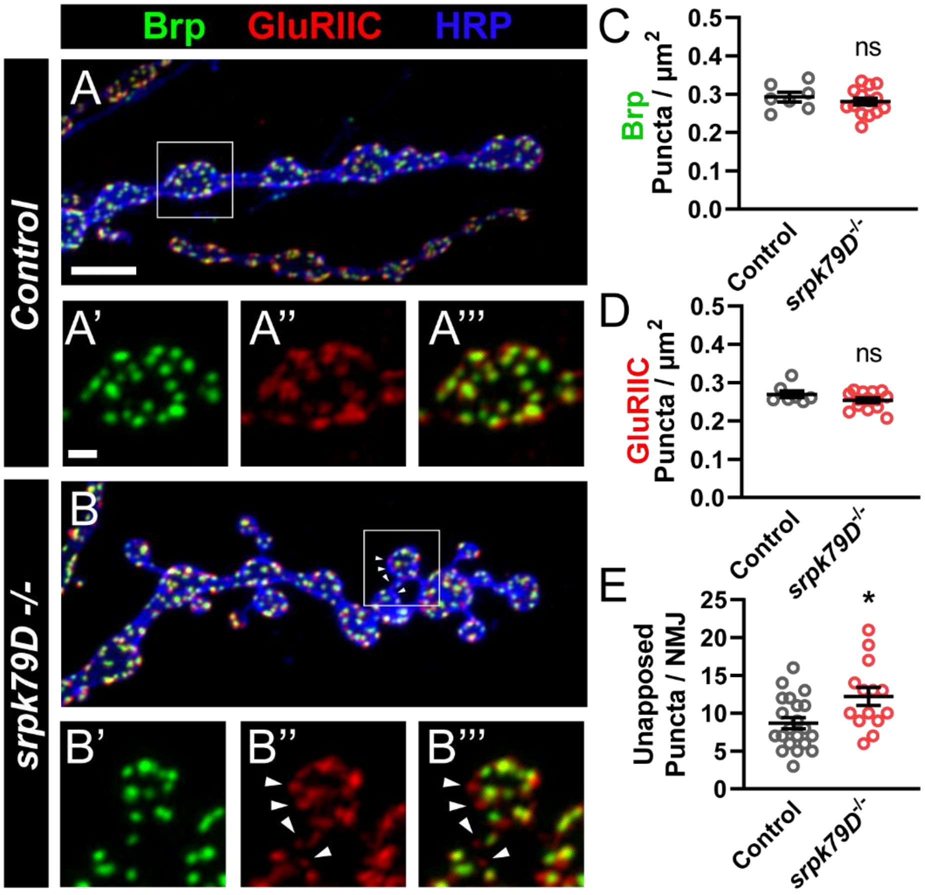

Fig. S10. Active zone / receptor apposition is disrupted following loss of *srpk79D*

(A-B) Representative confocal images from control (A) and *srpk79D* mutant (B) NMJs stained with antibodies to Brp (green), GluRIIC (red), and HRP (blue). Arrowheads in (B) indicate unapposed puncta. Scale = 5µm, 2µm (insets)

(C) Quantification of Brp density.

(D) Quantification of GluRIIC density.

(E) Quantification of the number of unapposed puncta per NMJ.

For all experiments, data are shown as mean ± SEM. * $p < 0.05$, *ns* = not significant. Significance was determined using a two-tailed Student's t-test. $n \geq 14$ NMJs, 8 larvae.

Development • Supplementary information

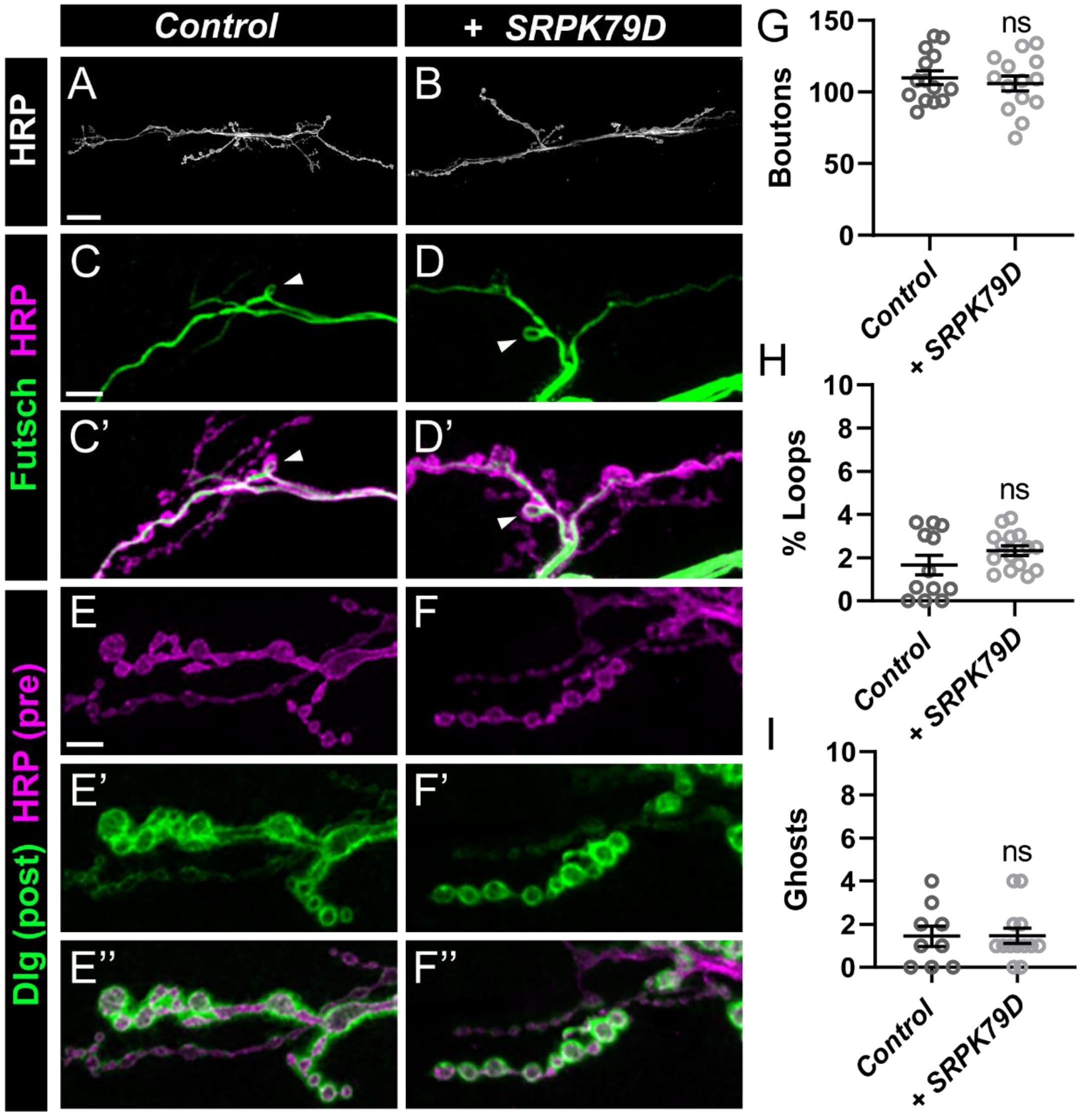

Fig. S11. Overexpression of SRPK79D alone does not affect synapse growth or maturation.

(A-B) Representative confocal images of NMJs from control (A) and motoneuron SRPK79D overexpression (B) larvae stained with antibodies to HRP. Scale = 25μm.

(C-D) Representative confocal images of NMJs from control (C) and motoneuron SRPK79D overexpression (D) larvae stained with antibodies to Futsch (green) and HRP (magenta). Arrows indicate Futsch loops. Scale = 5μm.

Development • Supplementary information

(E-F) Representative confocal images of NMJs from control (E) and motoneuron SRPK79D overexpression (F) larvae stained with antibodies to Dlg (green) and HRP (magenta). Scale = 5μm.

(G) Quantification of bouton number from A-B.

(H) Quantification of Futsch loops from C-D.

(I) Quantification of ghost boutons from E-F.

For all experiments, data are shown as mean ± SEM. *ns* = not significant. Significance was determined using a two-tailed Student's t-test. $n \geq 8$ NMJs, 4 larvae.

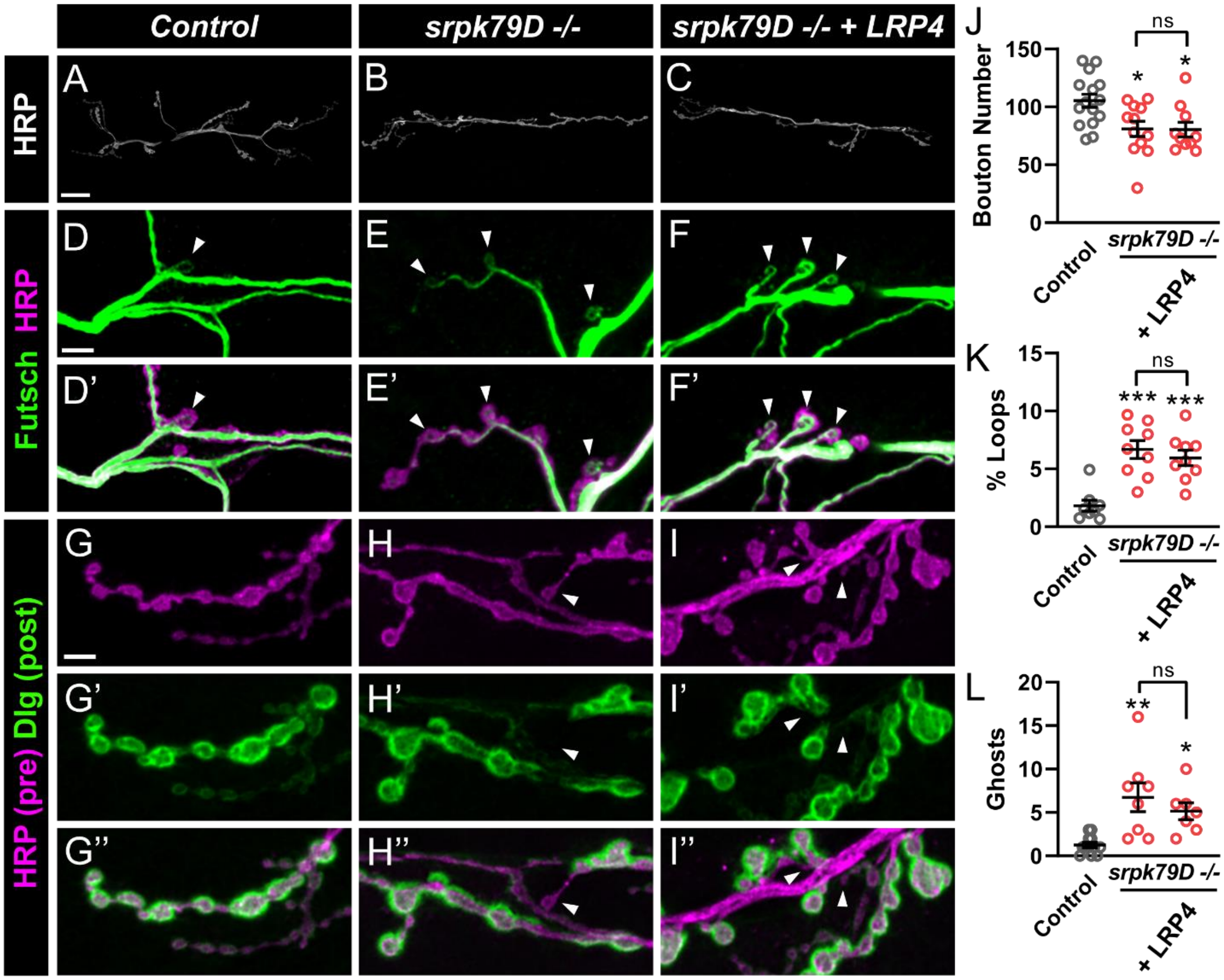

Fig. S12. Overexpression of *lrp4* is not sufficient to rescue *srpk79D* mutant phenotypes.

(A-C) Representative confocal images from control (A), *srpk79D* mutant (B) and *srpk79D* mutant expressing LRP4 in motoneuron (C) NMJs stained with antibodies to HRP. Scale = 25µm.

(D-F) Representative confocal images from control (D), *srpk79D* mutant (E) and *srpk79D* mutant expressing LRP4 in motoneuron (F) NMJs stained with antibodies to Futsch (green) and HRP (magenta). Arrows indicate Futsch loops. Scale = 5µm.

(G-I) Representative confocal images from control (G), *srpk79D* mutant (H) and *srpk79D* mutant expressing LRP4 in motoneuron (I) NMJs stained with antibodies to Dlg (green) and HRP (magenta). Arrows indicate ghost boutons. Scale = 5µm.

Development • Supplementary information

(J) Quantification of bouton number from A-C.

(K) Quantification of percent of boutons containing Futsch loops from D-F.

(L) Quantification of number of ghost boutons per NMJ from G-I.

For all experiments, data are shown as mean ± SEM. * $p < 0.05$, ** $p < 0.01$, *** $p < 0.001$, *ns* = not significant. Significance was calculated using one-way ANOVA, followed by Tukey's test for multiple comparisons. $n \geq 7$ NMJs, 4 larvae.

Table S1. Genotypes

Figure	Panel / Label	*Genotype(s)*
1	D-H	*w, LRP4-3xHA; +; +; +*
2	A, G	*+ / + or y; +; +; +*
	B, H	*w, lrp4*^{dalek} */ w, lrp4*^{dalek} *or y; +; +; +*
	K	*w / y; +; +; +*
	L	*w, lrp4*^{Del}*/ y; +; +; +*
3-4	Control	*+ / + or y; +; +; +*
	lrp4-/-	*w, lrp4*^{dalek} */ w, lrp4*^{dalek} *or y; +; +; +*
	+ N LRP4	*w, lrp4*^{dalek} */ w, lrp4*^{dalek} *or y; OK6-GAL4 / +; UAS-LRP4-HA / +; +*
	+ M LRP4	*w, lrp4*^{dalek} */ w, lrp4*^{dalek} *or y; +; mhc-GAL4 / UAS-LRP4-HA; +*
	N RNAi	*w, UAS-Dcr2 / + or y; OK6-GAL4 / UAS-LRP4-IR-108629; +; +*
	M RNAi	*w, UAS-Dcr2 / + or y; DMef2-GAL4 / UAS-LRP4-IR-108629; +; +*
5	A, C	*+ / + or y; +; +; +*
	B, D	*w, lrp4*^{dalek} */ w, lrp4*^{dalek} *or y; +; +; +*
6	A	*+ / + or y; +; +; +*
	B	*w / w or y; +; srpk79D*^{atc}*; +*
	C	*w, lrp4*^{dalek} */ w, lrp4*^{dalek} *or y; +; +; +*
	D	*w, lrp4*^{dalek} */ w, lrp4*^{dalek} *or y; +; srpk79D*^{atc}*; +*
7	A	*+ / + or y; +; +; +*
	B	*w, lrp4*^{dalek} */ w, lrp4*^{dalek} *or y; OK6-GAL4 / UAS-mCD8-GFP; +; +*
	C	*w, lrp4*^{dalek} */ w, lrp4*^{dalek} *or y; OK6-GAL4 / +; UAS-venus-SRPK79D; +*
8	A	*+ / + or y; +; +; +*
	B	*w, lrp4*^{dalek} */ w, lrp4*^{dalek} *or y; +; +; +*
	C	*w, lrp4*^{dalek} */ w, lrp4*^{dalek} *or y; OK6-GAL4 / +; UAS-venus-SRPK79D; +*
S1	A	*w / w or y; UAS-mCD8-GFP / + ; P{GMR90B08-GAL4}attP2 / + ; +*
	B	*w, C155-GAL4 / + or y; +; UAS-LRP4-HA / +; +*
	C	*w / w or y; + ; P{GMR90B08-GAL4}attP2 / UAS-LRP4-HA ; +*
S2	A	*+ / + or y; +; +; +*
	B	*w, LRP4-3xHA; +; +; +*
S4	A	*+ / + or y; +; +; +*
	B	*w, lrp4*^{dalek} */ w, lrp4*^{dalek} *or y; +; mhc-GAL4 / UAS-LRP4-HA; +*
	C	*w, UAS-Dcr2 / + or y; DMef2-GAL4 / UAS-LRP4-IR-108629; +; +*
S5	A	*w, lrp4*^{dalek} */ w, lrp4*^{dalek} *or y; +; +; +*
	B	*w, lrp4*^{dalek} */ w, lrp4*^{dalek} *or y; +; Repo-GAL4 / UAS-LRP4-HA; +*
S6	A	*w, C155-GAL4 / + or y; +; +; +*
	B	*w, C155-GAL4 / + or y; +; UAS-LRP4-HA / +; +*
S7	A	*+ / + or y; +; +; +*
	B	*w, lrp4*^{dalek} */ w, lrp4*^{dalek} *or y; +; mhc-GAL4 / UAS-LRP4-HA; +*
	C	*w, UAS-Dcr2 / + or y; DMef2-GAL4 / UAS-LRP4-IR-108629; +; +*
S9	A	*w; OK6-GAL4 / + or y; UAS-venus-SRPK79D / +; +*
S10	A	*+ / + or y; +; +; +*

	B	*w / w or y; +; srpk79D*^{atc}*; +*
S11	A	*+ / + or y; +; +; +*
	B	*w / w or y; OK6-GAL4 / +; UAS-venus-SRPK79D / +; +*
S12	A	*+ / + or y; +; +; +*
	B	*w / w or y; +; srpk79D*^{atc}*; +*
	C	*w / w or y; OK6-GAL4 / UAS-LRP4-HA; srpk79D*^{atc}*; +*

Development • Supplementary information

Table S2. ***n*** **values**

Panel	*n* (in NMJs, larvae) for 2C-F, M-O, 3, 4, 5, 6, 7, 8D-F<br>*n* (in boutons, larvae) for 2I, 8G					
	Control	*lrp4 -/-*				
2C	11, 7	12, 6				
2D	11, 7	12, 6				
2E	11, 7	12, 6				
2F	14, 7	12, 6				
2I	47, 8	60, 8				
2M	19, 9	12, 5				
2N	19, 9	12, 5				
2O	19, 9	12, 5				
	Control	*lrp4 -/-*	*+ N LRP4*	*+ M LRP4*	*N RNAi*	*M RNAi*
3I	15, 8	16, 8	16, 8	14, 8	16, 8	16, 8
3J	15, 8	15, 8	15, 8	13, 8	16, 8	16, 8
3K	15, 8	14, 8	15, 8	13, 8	16, 8	15, 8
4I	16, 8	16, 8	14, 7	16, 8	16, 8	16, 8
4J	14, 8	16, 8	16, 8	14, 8	15, 8	16, 8
	Control	lrp4 -/-				
5E	44, 4	46, 3				
5F	44, 4	46, 3				
5G	44, 4	46, 3				
5H	44, 4	46, 3				
5I	162, 4	133, 3				
	Control	*srpk79D -/-*	*lrp4 -/-*	*lrp4 -/- srpk79D -/-*		
6Q	15, 8	16, 8	14, 8	13, 8		
6R	15, 8	16, 8	16, 8	14, 8		
6S	14, 8	16, 8	16, 8	12, 6		
6T	8, 4	8, 4	7, 4	8, 4		
	Control	*lrp4 -/-+GFP*	*lrp4 -/- +SRPK79D*			
7M	8, 4	15, 8	16, 8			
7N	12, 8	7, 4	16, 8			
7O	10, 6	15, 8	16, 8			
7P	16, 8	16, 8	16, 8			
	Control	*lrp4 -/-*	*lrp4 -/- + SRPK79D*			
8D	8, 4	8, 4	7, 4			
8E	8, 4	8, 4	7, 4			
8F	8, 4	7, 4	8, 4			
8G	21, 4	17, 4	16, 4			

Development • Supplementary information
