# Supplementary material for: Neuronal LRP4 directs the development, maturation and cytoskeletal organization of Drosophila peripheral synapses
Source: Development. 2024 Jun 3;151(11):dev202517. doi: 10.1242/dev.202517 (PMC11190576; doi:10.1242/dev.202517)
Supplement: Supplementary file 1 [file develop-151-202517_review_history.pdf]

Neuronal LRP4 directs the development, maturation and cytoskeletal organization of *Drosophila* peripheral synapses

Alison T DePew, Joseph J Bruckner, Kate M O'Connor-Giles and Timothy J Mosca
DOI: 10.1242/dev.202517

Editor: Irene Miguel-Aliaga

Review timeline
Original submission: 9 November 2023
Editorial decision: 14 December 2023
First revision received: 5 April 2024
Accepted: 2 May 2024

Original submission

First decision letter

MS ID#: DEVELOP/2023/202517

MS TITLE: Neuronal LRP4 directs the development, maturation, and cytoskeletal organization of peripheral synapses

AUTHORS: Alison T DePew, Joseph J Bruckner, Kate M O'Connor-Giles, and Timothy J Mosca

I have now received all the referees' reports on the above manuscript, and have reached a decision. The referees' comments are appended below, or you can access them online: please go to BenchPress and click on the 'Manuscripts with Decisions' queue in the Author Area.

As you will see, the referees express considerable interest in your work, but have some significant criticisms and recommend a substantial revision of your manuscript before we can consider publication. If you are able to revise the manuscript along the lines suggested, which may involve further experiments, I will be happy receive a revised version of the manuscript. Your revised paper will be re-reviewed by one or more of the original referees, and acceptance of your manuscript will depend on your addressing satisfactorily the reviewers' major concerns. Please also note that Development will normally permit only one round of major revision. If it would be helpful, you are welcome to contact us to discuss your revision in greater detail. Please send us a point-by-point response indicating your plans for addressing the referees' comments, and we will look over this and provide further guidance.

Please attend to all of the reviewers' comments and ensure that you clearly highlight all changes made in the revised manuscript. Please avoid using 'Tracked changes' in Word files as these are lost in PDF conversion. I should be grateful if you would also provide a point-by-point response detailing how you have dealt with the points raised by the reviewers in the 'Response to Reviewers' box. If you do not agree with any of their criticisms or suggestions please explain clearly why this is so.

Reviewer 1

Advance summary and potential significance to field

© 2024. Published by The Company of Biologists under the terms of the Creative Commons Attribution License (https://creativecommons.org/licenses/by/4.0/).

Not only it provides genetic evidence that LRP4 regulates presynaptic development in a cell-autonomous manner, but also (and importantly) it reveals a downstream effector of LRP4 (i.e., SRPK79D).

Comments for the author

LRP4 is key to mammalian NMJ formation and maintenance and in mammalian brains regulates synapse development and function by regulating astrocytes. In Drosophila, Mosca and colleagues earlier showed that LRP4 acts presynaptically for olfactory synapses. The current study, genetic evidence has been provided that LRP4 is expressed in motoneurons, and is required for synaptic development.
It regulates presynaptic assembly in a cell-autonomous manner, but postsynaptic development in a cell non-autonomous manner. The authors used elegant genetic experiments to show that LRP4 acts via the downstream SR protein kinase (SRPK79D) in motoneurons.
The study addresses an important question in synapse development. Not only it provides genetic evidence that LRP4 regulates presynaptic development in a cell-autonomous manner, but also (and importantly) it reveals a downstream effector of LRP4 (i.e., SRPK79D). Experiments were well designed and executed and data of high quality. I believe that the paper would be of great interest to people in the field and in neural development.
However, I believe it important to be clear regarding how LRP4 regulates synapse formation in Drosophila and in vertebrates. Although “presynaptic LRP4-dependent mechanisms” exist in “invertebrate and vertebrate synapse formation”, the mechanisms are quite different. As shown by the last author of this study, in Drosophila, LRP4 functions as a presynaptic molecule for presynaptic assembly; however, at mammalian NMJs, LRP4 is located mainly if not exclusively at the postsynaptic terminals for NMJ formation including presynaptic differentiation. There is no genetic evidence that in mammals, LRP4 functions as a presynaptic molecule at the NMJ or in the CNS. Recent scRNA-seq data indicate that LRP4 is almost exclusively expressed in a subset of astrocytes, not at all in neurons. Therefore, it is confusing to state “a similar synaptogenic role for LRP4 exists in the mammalian central brain…” or “whether LRP4 function in the mammalian CNS is pre- or postsynaptic is not known…”. If papers by Gomez 2014, Handara 2019 and Tian 2006 must be cited, I would like to suggest to point out exactly under what conditions the conclusions were drawn (like in vitro, overexpressing, etc.). Otherwise, the field remains muddy. Notice that the fact that LRP4 acts postsynaptically at mammalian NMJs or in CNS astrocytes by no means diminishes the significance of LRP4’s presynaptic role in Drosophila and finding how it works (such as via SRPK79D). Instead, it makes the studies of fly LRP4 more interesting and revealing.
Therefore, I would suggest that multiple places (title, abstract, discussion) in the manuscript be revised. For the same reasons, the title should indicate that LRP4 here is neuronal LRP4 in Drosophila, not vertebrates.
Dok7 discovery and interaction with MuSK were not properly credited. The authors cited Berganmin et al., 2010.
LRP4 gain of function has been shown to slow NMJ aging (Zhao et al.). A recent paper showed patient anti-LRP4 antibodies damage the NMJ.

<u>Reviewer 2</u>

Advance summary and potential significance to field

see below

Comments for the author

In this manuscript by DePew et al the authors report on a previously underappreciated role for the cell surface receptor LRP4 in directing presynaptic development at the Drosophila NMJ. The authors use a powerful combination of mutant analysis, confocal imaging, electrophysiology, and electron microscopy to characterize various roles of Lrp4 in drosophila NMJ development. Moreover, the authors identify the SR- protein kinase SRPK79D as a downstream target through which LRP4 promotes NMJ development. The experiments are well designed and executed, and for the most part the authors’ conclusions are well supported by experimental evidence. While the results are very compelling, they stand somewhat in isolation without a clear mechanism or even working

© 2024. Published by The Company of Biologists under the terms of the Creative Commons Attribution License (https://creativecommons.org/licenses/by/4.0/).

model of how Lrp4/ and SRPK79D promote presynaptic development. While the authors seem to favor the idea that Lrp4 acts as an organizer of multiple processes during presynaptic development, experimental evidence for such role is somewhat weak. While they nicely characterize multiple presynaptic defects in Lrp4 mutants, it is unclear whether these phenotypes reflect - as the authors favor- multiple independent presynaptic roles for Lrp4, or instead stem from a common root ie are consequences of a single early defect – e.g. in cytoskeletal organization. Teasing this apart e.g. by activating or inactivating lrp4 at slightly different timepoints would greatly support the idea that Lr4 acts as an organizer of presynaptic differentiation and significantly increase the impact of the study.

Minor points :

1. The specific n's for individual groups within experiments is not provided, and instead is often listed as greater than/equal to. Some of the effect sizes are rather small so having the exact n for each condition listed seems important. This could be shown on the graphs with dots or just have the number listed in each bar on the bar graphs.

2. The data regarding the results of muscle-specific KD or rescued expression is not shown for either figure 3 & 4. Only quantification is shown, but more complete data for muscle-specific KD and rescue expression should be shown e.g. in supplement.

3. The manuscript would benefit from amore detailed discussion of how lrp4/ SRPK79D promotes presynaptic differentiation.

4. The authors conclude that their data suggests an 'essential' function for synaptic development. However, given that synapses do form - albeit incompletely- in Lrp4 mutants is more consistent with a critical but not essential role in synapse development. As such removing essential from the role of Lrp4 seems appropriate.

<u>Reviewer 3</u>

Advance summary and potential significance to field

The manuscript by DePew and colleagues examine LRP4, a conserved transmembrane protein implicated in clustering of postsynaptic acetylcholine receptors by bridging agrin and MuSK. The authors have previously demonstrated a presynaptic role for LRP4 in the Drosophila olfactory system and showed that LRP4 functions via the conserved kinase SRPK79D to ensure normal synapse number and behavior. In the current manuscript, the authors expand the analysis of the role of LRP4 in synapse organization using the larval NMJ system. They documented the LRP4 localization at synaptic terminals by generating a new, endogenously tagged variant. They performed a large battery of tests to examine the function of LRP4 at presynaptic locations using their previously published null allele.
Among the loss-of-function phenotypes, the authors report a disruption of the active zone organization that impacts the neurotransmitter release. Also, the mutant NMJs have fewer boutons, disrupted microtubule organization and reduced postsynaptic spectrin accumulation. The authors use genetics and epistasis experiments to test whether SRPK79D functions downstream LRP4 and found that overexpression of SRPK79D rescued some of the LRP4 loss-of-function phenotypes. The epistasis analysis is minimal and unlike in their previous paper, the authors stop short of connecting all the dots back to Brp and active zone integrity. In my view there is a figure missing at the end, examining whether overexpression of SRPK79D rescues the donut shape for the Brp puncta and the electrophysiological defects of lrp4 mutants. The authors discuss this possibility but do not investigate it. In the last three lines of the discussion, they also hint at a different LRP4 pool fulfilling this function, the glial LRP4. In any case, these data need to be acquired and shown, rather than alluded to in a "limitations of this study" section.

The manuscript brings some new information to what the authors reported before at CNS synapses (in particular, by documenting a role for presynaptic LRP4 in modulating microtubule dynamics) and expands our understanding of the role of LRP4 in synapse organization.

© 2024. Published by The Company of Biologists under the terms of the Creative Commons Attribution License (https://creativecommons.org/licenses/by/4.0/).

The data shown (except for EM) are of high quality. The manuscript is well organized and nicely written, with lengthy explanations introducing every new experiment. But there are some parts that are too long and should be consolidated (for example the introduction) before the manuscript could be considered for publication.

Comments for the author

Major concerns:

1) The introduction is too long and reads like a review. The authors should consolidate it by focusing on the relevant points. Parts of the discussion should be consolidated too. As is, it dilutes some of the strong points of the manuscript.

2) The authors conclude that LRP4 is an instructive signal that influences the number of boutons by regulating the microtubules organization. Also, they argue that LRP4 is the signal upstream SRPK79D.
The authors should test whether overexpression of LRP4 increases the number of boutons in an SRPK79D mutant background. This result will strengthen their epistasis arguments.

3) The manuscript needs one more figure examining whether overexpression of SRPK79D rescues the organization of the Brp-marked active zones and the electrophysiological defects of lrp4 mutants.
The results, even if negative, will be informative to the reader - primed here to look for modulators of active zone integrity and activity.
If the number of figures becomes a problem, the authors may consider moving the EM data in the supplementary material.

4) S1: What happens to LRP4-HA when overexpressed using the LRP4-Gal4 line? Do the authors find any LRP4 expression elsewhere in the muscle or glia?

Minor issues:
1) It will be very nice if the authors have a chance to improve the EM data. However, one understands that this technology is not readily accessible.
2) does SRPK79D require its kinase activity to rescue the LRP4 loss-of-function phenotypes?
3) 883- The authors write: "Note the ring-like structure of HA staining surrounding Brp puncta."- In the images presented here, I don't really see it. The authors may want to provide a 3D reconstitution of their STED images to better support this point.
4) 211- S1A-B should be S1A also, 224 - S1C-D should be S1B 5) S1: What happens to LRP4-HA when overexpressed in the muscle? Does it accumulate at the NMJ too?
Could the author speculate on what is upstream LRP4? Are there any cholinergic receptors involved?

First revision

<u>Author response to reviewers' comments</u>

Response to Reviewers

We thank the three expert reviewers for their thorough assessment of our manuscript. We appreciate their positive receipt of our work and acknowledgment of its importance to the field. Their helpful and insightful comments have considerably improved the rigor and readability of our work. The revised manuscript has two major additions to address the reviewers' concerns as well as multiple smaller changes. We first describe the two major additions and then provide a point-by-point response regarding all additional changes to the manuscript. We also endeavored to shorten the manuscript considerably, cutting over 4000 words from the original submission. We hope these additions are satisfactory to address the reviewer's concerns and make the manuscript appropriate for publication.

© 2024. Published by The Company of Biologists under the terms of the Creative Commons Attribution License (https://creativecommons.org/licenses/by/4.0/).

1. Our original manuscript demonstrated that SRPK79D overexpression in an *lrp4* mutant background suppressed growth, maturation, and cytoskeletal defects associated with LRP4 loss. We have now added an additional figure demonstrating that overexpression of SRPK79D in an *lrp4* mutant background is also sufficient to rescue the active zone / glutamate receptor apposition defects we observe (Figure 8). Interestingly, however, we find that SRPK79D overexpression does not suppress defects in active zone organization observed with super resolution microscopy, suggesting an LRP4-dependent / SRPK79D-independent aspect of individual active zone biogenesis.

2. To expand upon the genetic pathway involving LRP4 and SRPK79D, we performed additional experiments to test if overexpression of LRP4 is sufficient to rescue the phenotypes observed following loss of *srpk79D*. We find that LRP4 overexpression is not sufficient to rescue any of the phenotypes tested (bouton number, Futsch loops, and ghost boutons), providing further evidence to support our model wherein SRPK79D functions downstream of LRP4.

The following is a point-by-point response detailing the changes made to the manuscript to address specific reviewer comments:

Reviewer 1 Advance Summary and Potential Significance to Field:
Not only it provides genetic evidence that LRP4 regulates presynaptic development in a cell-autonomous manner, but also (and importantly) it reveals a downstream effector of LRP4 (i.e., SRPK79D).

Reviewer 1 Comments for the Author:
LRP4 is key to mammalian NMJ formation and maintenance and in mammalian brains, regulates synapse development and function by regulating astrocytes. In Drosophila, Mosca and colleagues earlier showed that LRP4 acts presynaptically for olfactory synapses. The current study, genetic evidence has been providedthat LRP4 is expressed in motoneurons and is required for synaptic development. It regulates presynaptic assembly in a cell-autonomous manner, but postsynaptic development in a cell non-autonomous manner. The authors used elegant genetic experiments to show that LRP4 acts via the downstream SR protein kinase (SRPK79D) in motoneurons.

The study addresses an important question in synapse development. Not only it provides genetic evidence that LRP4 regulates presynaptic development in a cell-autonomous manner, but also (and importantly) it reveals a downstream effector of LRP4 (i.e., SRPK79D). Experiments were well designed and executed and data of high quality. I believe that the paper would be of great interest to people in the field and in neural development.

We thank the reviewer for their kind comments and appreciation of our manuscript.

However, I believe it important to be clear regarding how LRP4 regulates synapse formation in Drosophila and in vertebrates. Although “presynaptic LRP4-dependent mechanisms” exist in “invertebrate and vertebrate synapse formation”, the mechanisms are quite different. As shown by the last author of this study, in Drosophila, LRP4 functions as a presynaptic molecule for presynaptic assembly; however, at mammalian NMJs, LRP4 is located mainly if not exclusively at the postsynaptic terminals for NMJ formation including presynaptic differentiation. There is no genetic evidence that in mammals, LRP4 functions as a presynaptic molecule at the NMJ or in the CNS. Recent scRNA-seq data indicate that LRP4 is almost exclusively expressed in a subset of astrocytes, not at all in neurons. Therefore, it is confusing to state “a similar synaptogenic role for LRP4 exists in the mammalian central brain…” or “whether LRP4 function in the mammalian CNS is pre- or postsynaptic is not known…”. If papers by Gomez 2014, Handara 2019 and Tian 2006 must be cited, I would like to suggest to point out exactly under what conditions the conclusions were drawn (like in vitro, overexpressing, etc.). Otherwise, the field remains muddy. Notice that the fact that LRP4 acts postsynaptically at mammalian NMJs or in CNS astrocytes by no means diminishes the significance of LRP4’s presynaptic role in Drosophila and finding how it works (such as via SRPK79D). Instead, it makes the studies of fly LRP4 more interesting and revealing.

© 2024. Published by The Company of Biologists under the terms of the Creative Commons Attribution License (https://creativecommons.org/licenses/by/4.0/).

We agree with the reviewer's assessment of the existing LRP4 literature and have updated our introduction to better reflect the known roles of LRP4 in the brain (line 107-114). We also thank the reviewer for their appreciation of non-mammalian findings!

Therefore, I would suggest that multiple places (title, abstract, discussion) in the manuscript be revised. For the same reasons, the title should indicate that LRP4 here is neuronal LRP4 in Drosophila, not vertebrates.

We adjusted the title to indicate these findings were discovered in *Drosophila*.

Dok7 discovery and interaction with MuSK were not properly credited. The authors cited Bergamin et al., 2010.

We corrected the missing citations for Dok7 discovery and interaction (line 93).

LRP4 gain of function has been shown to slow NMJ aging (Zhao et al.). A recent paper showed patient anti-LRP4 antibodies damage the NMJ.

We included an additional citation (line 99) to reflect recent work on the role of LRP4 in NMJ aging.

Reviewer 2 Comments for the Author:

In this manuscript by DePew et al the authors report on a previously underappreciated role for the cell surface receptor LRP4 in directing presynaptic development at the Drosophila NMJ. The authors use a powerful combination of mutant analysis, confocal imaging, electrophysiology, and electron microscopy to characterize various roles of Lrp4 in drosophila NMJ development. Moreover, the authors identify the SR- protein kinase SRPK79D as a downstream target through which LRP4 promotes NMJ development. The experiments are well designed and executed, and for the most part the authors' conclusions are well supported by experimental evidence. While the results are very compelling, they stand somewhat in isolation without a clear mechanism or even working model of how Lrp4/ and SRPK79D promote presynaptic development. While the authors seem to favor the idea that Lrp4 acts as an organizer of multiple processes during presynaptic development, experimental evidence for such role is somewhat weak. While they nicely characterize multiple presynaptic defects in Lrp4 mutants, it is unclear whether these phenotypes reflect - as the authors favor- multiple independent presynaptic roles for Lrp4, or instead stem from a common root i.e. are consequences of a single early defect – e.g. in cytoskeletal organization. Teasing this apart e.g. by activating or inactivating lrp4 at slightly different timepoints would greatly support the idea that Lr4 acts as an organizer of presynaptic differentiation and significantly increase the impact of the study.

We thank the reviewer for their thoughtful assessment of our work and the helpful comments for improving our manuscript. We agree with the reviewer's assertion that it remains unknown if LRP4 functions by influencing multiple independent cellular processes or whether the phenotypes observed stem from a common etiology. Indeed, our assessment of the *lrp4* mutant reveals a comprehensive constellation encompassing growth, cytoskeletal organization, active zone organization, and maturation. These phenotypes are rarely encountered concurrently, suggesting some level of independence between their cellular processes as opposed to relying on a single early defect, like cytoskeletal organization as the reviewer suggests. We favor the former explanation, though we fully acknowledge that the latter is a valid possibility. To explore this issue, and at the reviewer's interesting suggestion, we performed additional experiments to disrupt LRP4 function "mid-development" using temperature sensitive GAL80 in conjunction with GAL4/UAS to provide temporal regulation of an LRP4-RNAi transgene (Response to Reviewers Figure 1). We allowed larvae to develop with normal LRP4 function during embryogenesis and the first instar stage (permissive temperature) before shifting to a restrictive temperature that activated *lrp4* RNAi, causing the larvae to complete the second and third instar stages with *lrp4* impaired. Given the available reagents and methodology, this was the best way to engage the reviewer's suggestion. We assessed multiple phenotypes (bouton number, ghost boutons, and cytoskeletal Futsch organization) and found that bouton number and microtubule organization were unaffected with later *lrp4* knockdown, but that synaptic maturation (observed via an increase in ghost boutons) was still notably impaired. On the surface, these results suggest that the diverse phenotypes observed following loss of *lrp4* do not result from a common underlying cause like cytoskeletal organization

© 2024. Published by The Company of Biologists under the terms of the Creative Commons Attribution License (https://creativecommons.org/licenses/by/4.0/).

because we observe maturation defects without accompanying microtubule defects. The data also suggest that LRP4 may function early in development to mediate its effects and is largely dispensable for synaptic maturation. We present the data here in the revision, however, for two major reasons. First, given the imprecise nature of this temperature-shifting strategy and the timing needed to engage the RNAi response *in vivo*, we cannot be certain that sufficient LRP4 knockdown has occurred by the time the phenotypes are assayed at the late third instar stage. Second, maturation necessarily occurs later compared to bouton growth and presynaptic cytoskeletal organization so the persistence of a maturation phenotype may simply reflect the temporal nature of the event rather than a separable phenotype. As a result, we do not feel the data provide an unambiguous assessment of the question and thus the data do not rise to the level of rigor that we would like to see in the full manuscript. We invite the reviewer to assess the data here, however, and would be glad to continue the conversation.

NOTE: We have removed unpublished data that had been provided for the referees in confidence.

Minor points:

1. The specific n's for individual groups within experiments is not provided, and instead is often listed as greater than/equal to. Some of the effect sizes are rather small so having the exact n for each condition listed seems important. This could be shown on the graphs with dots or just have the number listed in each bar on the bar graphs.

Thank you for the suggestion! To better illustrate the specific *n* values for individual groups, we have changed the format of the graphs to show individual data points. We have also included a table with the exact *n* value for each condition.

2. The data regarding the results of muscle-specific KD or rescued expression is not shown for either figure 3 & 4. Only quantification is shown, but more complete data for muscle-specific KD and rescue expression should be shown e.g. in supplement.

We have included additional supplemental figures (Fig.S4 and Fig.S7) with representative images for the muscle data shown in figures 3 and 4.

3. The manuscript would benefit from a more detailed discussion of how lrp4/ SRPK79D promotes presynaptic differentiation.

We have updated the Discussion with additional details of how *lrp4* / *srpk79D* might promote presynaptic differentiation (line 518-523).

4. The authors conclude that their data suggests an 'essential' function for synaptic development. However, given that synapses do form - albeit incompletely- in Lrp4 mutants is more consistent with a critical but not essential role in synapse development. As such removing essential from the role of Lrp4 seems appropriate.

We agree with the author's assessment of LRP4 as a molecule critical for synapse development and have removed our use of the word "essential" to reflect our findings more appropriately (lines 224, 492, 495).

Reviewer 3 Advance Summary and Potential Significance to Field:

The manuscript by DePew and colleagues examine LRP4, a conserved transmembrane protein implicated in clustering of postsynaptic acetylcholine receptors by bridging agrin and MuSK. The authors have previously demonstrated a presynaptic role for LRP4 in the Drosophila olfactory system and showed that LRP4 functions via the conserved kinase SRPK79D to ensure normal synapse number and behavior. In the current manuscript, the authors expand the analysis of the role of LRP4 in synapse organization using the larval NMJ system. They documented the LRP4 localization at synaptic terminals by generating a new, endogenously tagged variant. They performed a large battery of tests to examine the function of LRP4 at presynaptic locations using their previously published null allele. Among the loss-of-function phenotypes, the authors report a disruption of the active zone organization that impacts the neurotransmitter release. Also, the mutant NMJs have

© 2024. Published by The Company of Biologists under the terms of the Creative Commons Attribution License (https://creativecommons.org/licenses/by/4.0/).

fewer boutons, disrupted microtubule organization and reduced postsynaptic spectrin accumulation. The authors use genetics and epistasis experiments to test whether SRPK79D functions downstream LRP4 and found that overexpression of SRPK79D rescued some of the LRP4 loss-of-function phenotypes. The epistasis analysis is minimal and, unlike in their previous paper, the authors stop short of connecting all the dots back to Brp and active zone integrity. In my view there is a figure missing at the end, examining whether overexpression of SRPK79D rescues the donut shape for the Brp puncta and the electrophysiological defects of lrp4 mutants. The authors discuss this possibility but do not investigate it. In the last three lines of the discussion, they also hint at a different LRP4 pool fulfilling this function, the glial LRP4. In any case, these data need to be acquired and shown, rather than alluded to in a “limitations of this study” section.

The manuscript brings some new information to what the authors reported before at CNS synapses (in particular, by documenting a role for presynaptic LRP4 in modulating microtubule dynamics) and expands our understanding of the role of LRP4 in synapse organization. The data shown (except for EM) are of high quality. The manuscript is well organized and nicely written, with lengthy explanations introducing every new experiment. But there are some parts that are too long and should be consolidated (for example the introduction) before the manuscript could be considered for publication.

We thank the reviewer for their thorough assessment of our manuscript and insightful suggestions. We have performed multiple experiments to address these concerns. We also appreciate the reviewer’s interest in a glial role for LRP4. We performed additional experiments to more explicitly examine a potential role for glial LRP4 function in synaptic development (Fig.S5). In addition to data shown in Figures 3 and 4 demonstrating complete rescue of the *lrp4* mutant phenotypes following neuron-specific expression of LRP4, we included additional data to show that glial expression of LRP4 fails to rescue the bouton number phenotype observed following loss of *lrp4*. Since we find that LRP4 expression at the NMJ is limited to motor neurons, and only neuronal LRP4 expression is sufficient to rescue mutant phenotypes, we conclude that glial LRP4 does not contribute to the synapse development pathway described here. We have also adjusted the “Limitations of This Study” section to reflect these findings (line 561-563).

Reviewer 3 Comments for the Author:

Major concerns:

1) The introduction is too long and reads like a review. The authors should consolidate it by focusing on the relevant points. Parts of the discussion should be consolidated too. As is, it dilutes some of the strong points of the manuscript.

We thank the reviewer for this suggestion. We have significantly consolidated our introduction and discussion to relevant points to improve the readability of our manuscript.

2) The authors conclude that LRP4 is an instructive signal that influences the number of boutons by regulating the microtubules organization. Also, they argue that LRP4 is the signal upstream SRPK79D. The authors should test whether overexpression of LRP4 increases the number of boutons in an SRPK79D mutant background. This result will strengthen their epistasis arguments.

We performed additional experiments to test if overexpression of LRP4 in an *srpk79D* mutant background is sufficient to rescue the observed phenotypes (Fig.S12). We find that LRP4 expression is not sufficient to rescue defects in bouton number, microtubule organization, or maturation. This strongly suggests that LRP4 acts upstream of SRPK79D and we agree that the inclusion of this new data greatly strengthens our epistasis arguments.

3) The manuscript needs one more figure examining whether overexpression of SRPK79D rescues the organization of the Brp-marked active zones and the electrophysiological defects of lrp4 mutants. The results, even if negative, will be informative to the reader - primed here to look for modulators of active zone integrity and activity. If the number of figures becomes a problem, the authors may consider moving the EM data in the supplementary material.

© 2024. Published by The Company of Biologists under the terms of the Creative Commons Attribution License (https://creativecommons.org/licenses/by/4.0/).

We wholeheartedly agree that additional experiments examining whether overexpression of the downstream kinase, SRPK79D, is sufficient to rescue defects in active zone organization and function would greatly strengthen our understanding of this pathway. Unfortunately, we are not currently equipped to perform the requested electrophysiological experiment and the author who performed the original experiments in our collaborator lab has since left the lab, so we cannot provide that data. We did, however, perform additional experiments to assess active zone apposition via confocal microscopy and individual active zone organization via super-resolution STED microscopy. Gratifyingly, we find that the apposition defects observed in the *lrp4* mutant can be rescued by overexpression of SRPK79D and have included a new figure (Figure 8A-F) with these data. Intriguingly, however, we also found that SRPK79D overexpression does not suppress the active zone organization defects observed following loss of *lrp4* (Figure 8G) via STED, suggesting an SRPK79D-independent role for LRP4 in individual active zone organization. We hope the reviewer will agree that these are informative and important additions to the manuscript aimed at addressing SRPK79D roles in active zone apposition and organization.

4) S1: What happens to LRP4-HA when overexpressed using the LRP4-Gal4 line? Do the authors find any LRP4 expression elsewhere in the muscle or glia?

We have included additional data demonstrating that LRP4-HA expression is restricted to motor neurons at the NMJ when expressed using the LRP4-GAL4 line (Fig.S1C) and that we do not observe any significant muscle or glial expression above background.

Minor issues:

1) It will be very nice if the authors have a chance to improve the EM data. However, one understands that this technology is not readily accessible.

We acknowledge the reviewer's assessment of the quality of our EM data and appreciate the understanding that EM technology is not readily available. We have re-examined our dataset and endeavored to use better quality images where possible. We have also re-analyzed the data and find identical conclusions so we are confident that the current dataset is of sufficient validity to produce high quality results with significant rigor.

2) does SRPK79D require its kinase activity to rescue the LRP4 loss-of-function phenotypes?

We appreciate the reviewer's interest in whether SRPK79D requires its kinase activity to rescue *lrp4* mutant phenotypes. We agree that this is an interesting question and further investigation into SRPK79D kinase activity would greatly inform our understanding of this pathway. Unfortunately, we were unable to obtain previously made "kinase-dead" transgenes and could not conduct this experiment. Reconstruction of SRPK79D constructs that alter kinase activity would be an important future direction but we respectfully feel that it is outside of the scope of the current study.

3) 883- The authors write: "Note the ring-like structure of HA staining surrounding Brp puncta."- In the images presented here, I don't really see it. The authors may want to provide a 3D reconstitution of their STED images to better support this point.

We understand that the "ring-like structure" we note in our expression data may not be apparent. We have adjusted the language to reflect that HA-positive puncta are observed adjacent to active zones and provided a diagram to better reflect the expression pattern we observe (Fig1H).

NOTE: We have removed unpublished data that had been provided for the referees in confidence.

4) 211- S1A-B should be S1A also, 224 - S1C-D should be S1B

We thank the reviewer for noting this error and have adjusted the figure labels for figure S1.

5) S1: What happens to LRP4-HA when overexpressed in the muscle? Does it accumulate at the NMJ too? Could the author speculate on what is upstream LRP4? Are there any cholinergic receptors involved?

© 2024. Published by The Company of Biologists under the terms of the Creative Commons Attribution License (https://creativecommons.org/licenses/by/4.0/).

We appreciate the reviewer's interest in LRP4 expression. We have included additional data (Response to Reviewers Figure 2) to show LRP4-HA overexpressed in muscle, where we find it accumulates in muscle tissue, especially at membranes, including postsynaptically. Though interesting, this enrichment is often seen with non-specific membrane proteins (including membrane targeted GFP, mCD8-GFP), consistent with NMJ boutons being surrounded by the membrane-fold rich subsynaptic reticulum. As we do not see muscle expression above background, we believe this is an artifact of LRP4 expression and the presence of a transmembrane domain. Regarding speculation of elements upstream of LRP4, we do not think there are cholinergic receptors involved as these are not expressed at the fly NMJ. Ideally, the important identity of upstream LRP4 interacting proteins would be the ligand for LRP4. This is a major avenue of research in the lab but at this point, it would be too early to speculate on the identity of any potential ligands. We have adjusted our discussion (line 520) noting this as a question of interest for further investigation.

Second decision letter

MS ID#: DEVELOP/2023/202517

MS TITLE: Neuronal LRP4 directs the development, maturation, and cytoskeletal organization of Drosophila peripheral synapses

AUTHORS: Alison T DePew, Joseph J Bruckner, Kate M O'Connor-Giles, and Timothy J Mosca
ARTICLE TYPE: Research Article

Apologies for the delay in getting back to you, but one of the reviews has yet to materialise despite several reminders.

I am, however, happy to make a decision based on the two reviews that we have received. I am pleased to say that your manuscript has been accepted for publication in Development, pending our standard ethics checks.

Reviewer 1

Advance summary and potential significance to field

The paper revealed novel functions and potential mechanisms of presynaptic LRP4 in synapse formation.

Comments for the author

The authors have addressed my concerns and other reviewers. The manuscript has been improved. I support its publication.

Reviewer 2

Advance summary and potential significance to field

The authors have identified a new role for Lrp4 in directing/coordinating the development, maturation, an cytoskeletal organization of Drosophila peripheral synapses.

Comments for the author

The authors have done a very nice and thorough job of addressing each of this reviewer's comments

© 2024. Published by The Company of Biologists under the terms of the Creative Commons Attribution License (https://creativecommons.org/licenses/by/4.0/).
